# Orientation-dependent *Dxz4* contacts shape the 3D structure of the inactive X chromosome

**DOI:** 10.1038/s41467-018-03694-y

**Published:** 2018-04-13

**Authors:** G. Bonora, X. Deng, H. Fang, V. Ramani, R. Qiu, J. B. Berletch, G. N. Filippova, Z. Duan, J. Shendure, W. S. Noble, C. M. Disteche

**Affiliations:** 1 Genome Sciences, University of Washington, Seattle, WA 98195 USA; 2 Pathology, University of Washington, Seattle, WA 98195 USA; 3 Institute for Stem Cell and Regenerative Medicine, University of Washington, Seattle, WA 98195 USA; 4 Hematology, University of Washington, Seattle, WA 98195 USA; 5 Computer Science and Engineering, University of Washington, Seattle, WA 98195 USA; 6 Medicine, University of Washington, Seattle, WA 98195 USA

## Abstract

The mammalian inactive X chromosome (Xi) condenses into a bipartite structure with two superdomains of frequent long-range contacts, separated by a hinge region. Using Hi-C in edited mouse cells with allelic deletions or inversions within the hinge, here we show that the conserved *Dxz4* locus is necessary to maintain this bipartite structure. *Dxz4* orientation controls the distribution of contacts on the Xi, as shown by a massive reversal in long-range contacts after *Dxz4* inversion. Despite an increase in CTCF binding and chromatin accessibility on the Xi in *Dxz4*-edited cells, only minor changes in TAD structure and gene expression were detected, in accordance with multiple epigenetic mechanisms ensuring X silencing. We propose that *Dxz4* represents a structural platform for frequent long-range contacts with multiple loci in a direction dictated by the orientation of its bank of CTCF motifs, which may work as a ratchet to form the distinctive bipartite structure of the condensed Xi.

## Abstract (web summary)

The inactive X chromosome condenses into a bipartite structure. Here the authors use cells with allelic deletions or inversions to show that the *Dxz4* locus is necessary to maintain the bipartite structure and that *Dxz4* orientation controls the distribution of contacts on the inactive X chromosome.

## Introduction

Mammalian X chromosome inactivation (XCI) results in the silencing of one of the two X chromosomes in female somatic cells. Silencing is initiated by the expression of the long non-coding RNA (lncRNA) *Xist* from the future inactive X chromosome (Xi), followed by epigenetic changes that, among others, include histone H3 tri-methylation at lysine 27 (H3K27me3) enrichment and DNA methylation at CpG islands^1,2^. The Xi acquires a distinctive condensed structure (Barr body) unlike that of the active X chromosome (Xa) or the autosomes, and it is often located at the nuclear periphery or adjacent to the nucleolus^3–5^.

Genome-wide chromosome conformation capture (Hi-C) studies in mammalian cells and tissues demonstrate that chromosomes are divided into two types of compartments, A and B, associated with open and closed chromatin, respectively^6^. In contrast, allelic contact maps for the condensed human and mouse Xi show two superdomains of contacts separated by a hinge, forming a characteristic bipartite three-dimensional (3D) structure^7–11^. Long-range contacts are frequent within each superdomain, but are not observed between them, with little evidence of A/B compartments as compared to the Xa or the autosomes. The hinge region is partially preserved between human and mouse, and contains the macrosatellite repeat locus *DXZ4/Dxz4* in both species^7–11^. The *DXZ4/Dxz4* loci encode lncRNAs and bind CCCTC-binding factor (CTCF) and components of the cohesin complex only on the Xi, while the loci are methylated on the Xa, preventing CTCF binding^12–16^.

CTCF and cohesin are two of the main organizers of nuclear structure^17–20^. Highly dynamic chromatin loops form by progressive extrusion of chromatin fibers through cohesin rings, which proceed until a boundary element (BE), such as CTCF, stalls loop formation and ultimately defines topologically associated domains (TADs)^21,22^. Convergent CTCF-binding motifs (i.e., facing each other) at the base of chromatin loops favor strong interactions and the inversion of CTCF sites can disrupt loop formation^11,23,24^. At the *DXZ4/Dxz4* loci CTCF motifs are arranged in tandem orientation, with an estimated 10–100 copies in human^14^ and 14 copies in mouse^12^. How the CTCF motif arrangement influences long-range chromatin contacts on the Xi is unknown. In addition to *Dxz4*, the mouse hinge region originally defined using Hi-C also contains the mouse-specific minisatellite repeat *Ds-TR* whose function is unknown^7,12^. Both *Dxz4* and *Ds-TR* loci bind nucleophosmin, an essential component of the nucleolus, and could represent a large nucleolus-associated domain that may help position the Xi near the nucleolus^7,15^.

Here to determine the role of each element of the hinge in the maintenance of the 3D structure of the mouse Xi in relation to its silencing and nuclear positioning in somatic cells, we use allele-specific CRISPR/Cas9 editing to induce deletions and inversions specifically targeted to the Xi. We test the effects of these modifications on the overall 3D structure of the Xi using in situ DNase Hi-C^25^. Allele-specific analyses are done to assess changes to the distribution of contacts and the TAD structure. We score these changes in relation to CTCF-binding profiles obtained by chromatin immunoprecipitation-sequencing (ChIP-seq) and to chromatin accessibility profiles obtained by ATAC-seq. We determine the effects of genomic alterations of the hinge on the size and position of the Xi relative to the nuclear periphery and the nucleolus. Finally, gene expression changes are measured by RNA-seq. We conclude that *Dxz4* alone is necessary for maintenance of the condensed structure of the Xi in mouse fibroblasts, and that the distribution of contacts on the Xi depends on *Dxz4* orientation.

## Results

### *Dxz4* is necessary for Xi integrity

To evaluate specific elements located within the hinge that separates superdomains of long-range interactions on the mouse Xi, we used allele-specific CRISPR/Cas9 editing in F1 hybrid Patski cells, in which skewed XCI and frequent species-specific polymorphisms allows the Xi (C57BL/6 (BL6)) to be distinguished from the Xa (*Mus spretus)*^7,26,27^. We isolated the following edited clones: two independent clones with a large 127 kb deletion of the hinge, including both *Dxz4* and *Ds-TR* (Del-hinge clone a and b); two independent clones with a 44 kb inversion of *Dxz4* (Inv-Dxz4 clone a and b); and single clones with either a 44 kb deletion of *Dxz4* alone (Del-Dxz4), a 37 kb deletion of *Ds-TR* alone (Del-Ds-TR), or a small 907 bp inversion of two of three CTCF-binding sites located at the 5′end of *Ds-TR* (Inv-5′ Ds-TR). Additionally, a single clone (Patski2-4) was derived from wild-type (WT) cells (Supplementary Fig. 1a; see Methods).

**Fig. 1:**
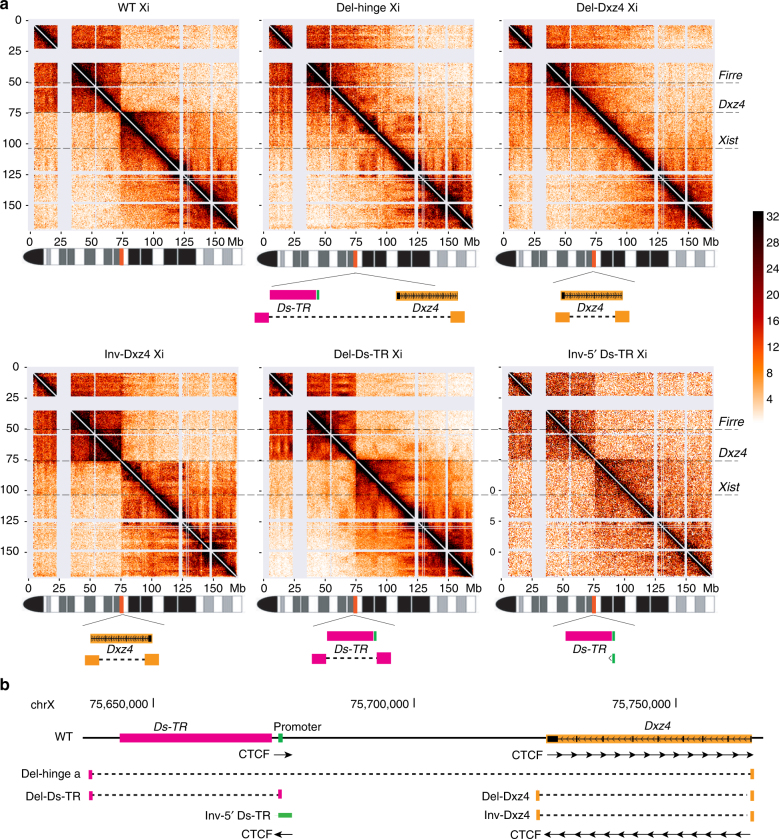
*Dxz4* alone is necessary to maintain the bipartite structure of the Xi. **a** Hi-C contact maps are shown at 500 kb resolution for the Xi in WT, Del-hinge (Xi deletion nt75637519–75764753), Del-Dxz4 (Xi deletion nt75721096–75764754), Inv-Dxz4 (Xi inversion nt75721096–75764754), Del-Ds-TR (Xi deletion nt75637501–75674037), and Inv-5′ Ds-TR (Xi inversion nt75674046–75674952). Note that fewer reads were obtained for the Inv-5′ Ds-TR, resulting in lower resolution (Supplementary Table 1). Allelic reads are listed in Supplementary Data 1. The location of *Dxz4*, *Firre*, and *Xist*, and schematics of the allele-specific deletions/inversions are shown. The color scale shows normalized contact counts. See Supplementary Fig. 2a for contact maps of the Xa in the same cell lines. **b** Relative position of the loci within the hinge region and location of the CRISPR/Cas9-induced alterations. Arrows indicate the orientation of CTCF motifs at *Dxz4*^12^ and at the 5′end of *Ds-TR*. See Supplementary Fig. 5b for CTCF binding at *Dxz4* on the Xi by ChIP-seq

In situ DNase Hi-C of Del-hinge clone a, Del-Dxz4, Inv-Dxz4 clone a, Del-Ds-TR, and Inv-5’ Ds-TR was performed in comparison to WT Patski cells (Supplementary Table 1)^7,25^. Similar numbers of allelic reads confirmed the presence of one Xa and one Xi (Supplementary Data 1), which was also verified by karyotyping and fluorescence in situ hybridization (FISH) analyses (see below and Methods). Both the large hinge deletion (Del-hinge) and the deletion of *Dxz4* alone (Del-Dxz4) dramatically disrupted the Xi bipartite structure, implicating *Dxz4* as the critical element in maintaining this structure (Fig. 1a, b). We noted subtle differences between Xi contact maps for the two deletions, with the Del-hinge map more closely resembling that of the Xa with respect to A/B compartment structure (Fig. 1a; Supplementary Fig. 2a). Thus, we cannot exclude that another hinge element plays a minor role in the Xi structure in the context of a *Dxz4* deletion, even though the deletion of *Ds-TR* (Del-Ds-TR) alone or inversion of its 5′end (Inv-5′ Ds-TR) did not cause any apparent disruption of the Xi bipartite structure. Inversion of *Dxz4* is also associated with persistence of the Xi bipartite structure, but caused extensive re-distribution of contacts as described below. There was no apparent change in contact maps for the Xa or the autosomes in any of the clones (Supplementary Fig. 2a).

We conclude that *Dxz4* alone is necessary for the formation of the two superdomains on the Xi.

### *Dxz4* contacts on the Xi are orientation-dependent

**Fig. 2:**
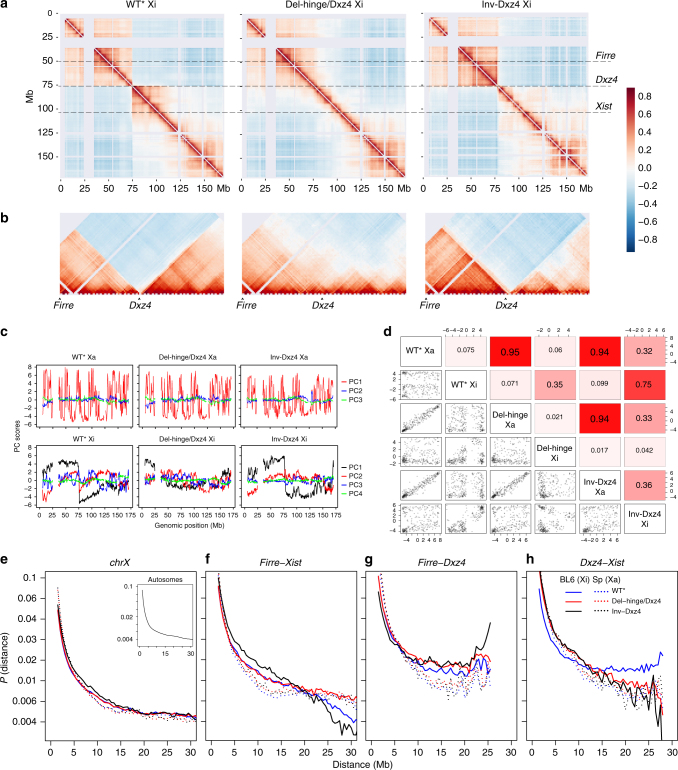
*Dxz4* deletion or inversion changes contact distribution on the Xi. **a** Contact maps (500 kb resolution) for the Xi in WT*, Del-hinge/Dxz4, and Inv-Dxz4 visualized using Pearson correlation to highlight contact probabilities. The color scale shows Pearson correlation values. **b** Pearson correlation-transformed contact maps (500 kb resolution) for 50 Mb around the *Dxz4* locus to highlight the loss of superdomain structure in Del-hinge/Dxz4 and the shift in contacts in Inv-Dxz4, where a new contact domain forms between *Firre* and *Dxz4*. The color scale shows Pearson correlation values. **c** Allelic principal component (PC) score profiles for the Xa and Xi chromosomes in WT*, Del-hinge/Dxz4, and Inv-Dxz4, based on distance-corrected, normalized contact maps with counts binned at 500 kb resolution. For the Xa, the top three allelic PC scores are shown in red, blue, and green, respectively. For the Xi, PC1 score profiles are plotted in black to emphasize that PC1 captures the bipartite structure rather than A/B compartments in WT*, and PC2-4 are shown in red, blue, and green, respectively. See Supplementary Fig. 3a for analysis of the variance in the PC score profiles of the X chromosomes and Supplementary Fig. 3b, c for analyses of autosomes. **d** Pair-wise Spearman correlation values and associated scatter plots between allelic PC1 scores for the Xa and Xi in WT*, Del-hinge/Dxz4, and Inv-Dxz4. Xi PC1 scores are less correlated between WT* and Del-hinge/Dxz4 (Spearman *ρ* = 0.35) compared to those of Xa (*ρ* = 0.95) and autosomes (*ρ* > 0.9). The Xi PC1 score in Inv-Dxz4 is less correlated with the WT* Xi PC1 profile (*ρ* = 0.75) than Xa PC1 scores (*ρ* = 0.94), indicative of changes in Xi-specific long-range contacts. See Supplementary Fig. 3d for analysis of autosomes. **e**–**h**. Plots of the average Hi-C interaction frequencies (at 500 kb resolution) as a function of genomic distance along the Xa (dotted line) and Xi (line) for WT* (blue), Del-hinge/Dxz4 (red), and Inv-Dxz4 (black) for the entire length of the X chromosomes (**e**) and for three regions along the X chromosome: *Firre-Xist* (~53 Mb, **f**); *Firre-Dxz4* (~25 Mb, **g**); *Dxz4-Xist* (~28 Mb, **h**). Inset: plots of average contacts as a function of distance for autosomes

Raw and Pearson correlation-transformed contact maps of the Xi for pooled datasets (Del-hinge/Dxz4 and WT*; see Methods) show that the hinge region almost disappears in Del-hinge/Dxz4, but not in Inv-Dzx4 (Fig. 2a; Supplementary Fig. 2b). Zooming in to examine a 50 Mb region around *Dxz4* confirms the loss of the boundary between superdomains in Del-hinge/Dxz4, and shows a strong shift in contacts in Inv-Dxz4, with new contacts appearing between *Dxz4* and *Firre*, a locus that also binds CTCF specifically on the Xi^15,28^ (Fig. 2b).

Changes to A/B compartment scores were evaluated by decomposition of allelic contact maps into principal components (PCs). PC score profiles for autosomes and for the Xa are very similar between cell lines with PC1 capturing A/B compartment structure (Supplementary Fig. 3a-d). In contrast, Xi PC1 captures the bipartite structure as a sign switch at *Dxz4* in WT*, but not in Del-hinge/Dxz4, resulting in a low correlation between these scores (Fig. 2c, d). Although a distinctive bipartite PC1 score profile is still evident along the Inv-Dxz4 Xi, its correlation with the WT* Xi PC1 profile is lower than that between WT* and Inv-Dxz4 Xa PC1 scores, indicative of changes in Xi-specific long-range contacts (Fig. 2c, d)

Strong differences in the contact decay curves (average contact counts as a function of distance) are evident for particular regions of the Xi between the different cell lines (Fig. 2e–h). Within the *Firre*-*Xist* region (~53 Mb), Del-hinge/Dxz4 shows a high proportion of very long-range interactions (>20 Mb), resulting in an Xa-like profile due to loss of insulation between superdomains, while Inv-Dxz4 makes even fewer very long-range interactions than WT*, implying that the inverted *Dxz4* locus results in even stronger insulation between superdomains than in its WT orientation (Fig. 2f). In the *Firre*-*Dxz4* region (~25 Mb) both *Dxz4*-edited cell lines display a high proportion of long-range contacts (10–20 Mb), with Inv-Dxz4 showing the highest proportion of very long-range contacts, reflecting increased contacts between *Dxz4* and *Firre* (Fig. 2g). In the *Dxz4*-*Xist* region (~28 Mb) there is a dramatic loss in long-range contacts in both Del-hinge/Dxz4 and Inv-Dxz4 (Fig. 2h). Note that a locus close to *Xist*, *x75* (chrX:105 037 350–105 070 119), previously shown to interact with *Dxz4* in mouse cells^8^, may also contribute to these regional effects.

**Fig. 3:**
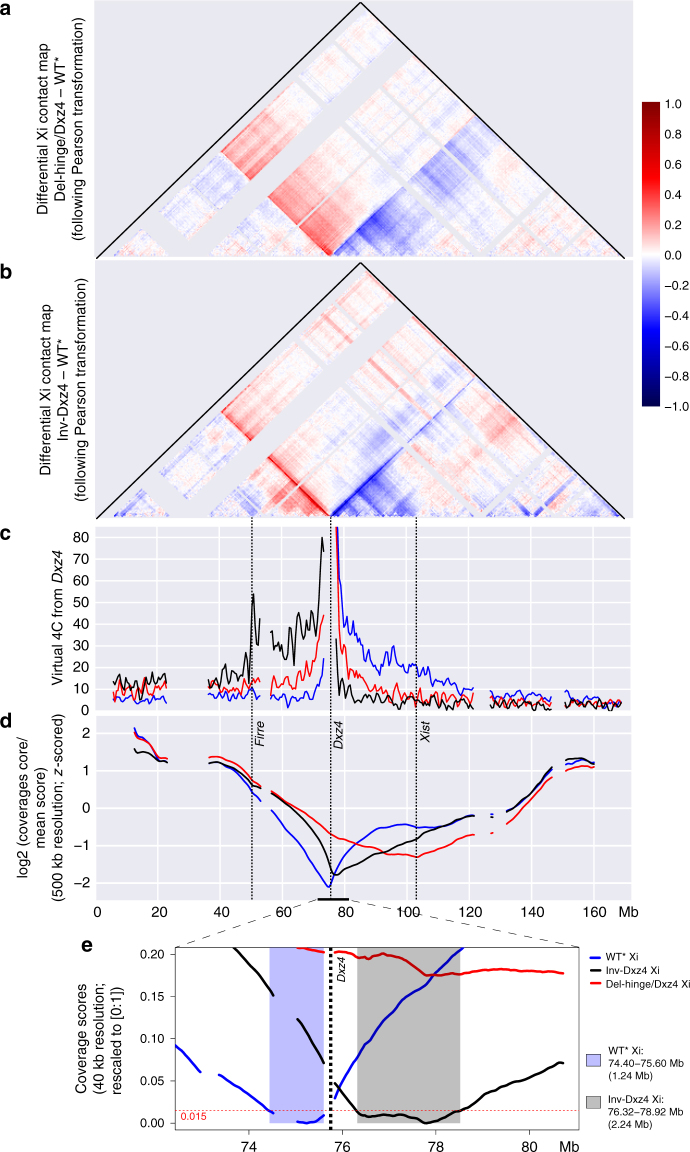
Unidirectional disruption of contacts on the Xi after *Dxz4* deletion or inversion. **a** Differential contact map based on Pearson correlation-transformed data at 500 kb resolution to highlight differences between Del-hinge/Dxz4 Xi and WT* Xi (loss or gain of contacts in the Del-hinge/Dxz4 versus WT* appear blue or red, respectively). The color scale shows differential Pearson correlation values. See Supplementary Fig. 3e, f for comparison with differential contact maps based on untransformed count data. **b** As in **a** to highlight differences between Inv-Dxz4 Xi and WT* Xi. See Supplementary Fig. 3g, h for comparison with differential contact maps based on untransformed count data. **c** Virtual 4C plots derived from Hi-C data at 500 kb resolution using a 500 kb region around *Dxz4* as the viewpoint on the Xi in WT* (blue), Del-hinge/Dxz4 (red), and Inv-Dxz4 (black). *Y*-axis (contact counts) limited to 20% of maximum. The positions of *Firre*, *Dxz4*, and *Xist* are indicated. See additional 4C analyses in Supplementary Fig. 3i. **d** Standardized coverage score profiles at 500 kb resolution for the Xi in WT* (blue), Del-hinge/Dxz4 (red), and Inv-Dxz4 (black). The positions of *Firre*, *Dxz4*, and *Xist* are indicated. See additional analysis of coverage scores for Xi and Xa in all cell lines in Supplementary Fig. 4a-d. **e** Coverage scores (rescaled to [0; 1]) at 40 kb resolution within a 8 Mb region around *Dxz4* for the Xi. The light blue background highlights the Xi boundary region of minimal interaction in WT* based on a threshold of 0.015 (horizontal red dashed line). The light gray background shows how this region is shifted to the right in Inv-Dxz4

Differential contact maps accentuate changes between cell lines (Fig. 3a, b; Supplementary Fig. 3e-h). Intriguingly, the differential contact map between Inv-Dxz4 and WT* shows that the *Dxz4* region makes many new contacts with centromeric loci, visualized as a red band running from *Dxz4* to the centromere, while losing interactions with telomeric loci (blue band; Fig. 3b). These bands (also known as “flames”^21,29^) represent the reversal of a line of unidirectional long-range contacts emanating from *Dxz4* due to the reversal in *Dxz4*’s orientation (Figs. 1a, 2b, and 3b). A subtle shift in the transition zone between the two superdomains is also discernable in these differential flames and will be described in more detail below. The unidirectional nature of interactions between *Dxz4* and other Xi loci is made further evident in virtual 4C plots based on Hi-C data using *Dxz4* as a viewpoint (Fig. 3c). Additional virtual 4C plots confirm that interactions between *Dxz4* and telomeric regions located as far as 40 Mb (chrX:75–115 Mb) are strongly reduced in Del-hinge/Dxz4 or Inv-Dxz4 (Supplementary Fig. 3i). The 4C plots show evidence of contact hotspots, for example, between *Dxz4* and either *Xist* or *x75* in WT*, and between *Dxz4* and *Firre* in Inv-Dxz4 (Fig. 3c; Supplementary Fig. 3i).

Contact frequency at all scales, including very long-range contacts, was quantified using a modified version of the coverage score measure^30^. As expected, the strong dip in coverage score at the boundary between superdomains in WT* disappears in Del-hinge/Dxz4, but is mostly retained in Inv-Dxz4 (Fig. 3d). Coverage scores reflect loss of contacts between *Dxz4* and telomeric regions in both Del-hinge/Dxz4 and Inv-Dxz4, and gain of contacts between the two superdomains specifically in Del-hinge/Dxz4, or between chrX:45–75 Mb in Inv-Dxz4 (Fig. 3d). Considering all edited clones, it is clear that changes in long-range contacts are only seen for deletions that include *Dxz4* and for *Dxz4* inversion (Supplementary Fig. 4a, b). Hierarchical clustering of coverage scores results in segregation of Xa’s from Xi’s, and within the Xi’s, segregation of Del-hinge, Del-Dxz4, and Inv-Dxz4 from the other mutants (Supplementary Fig. 4c, d). Coverage scores based on higher resolution (40 kb) data reveal that the region of minimal interactions between the two superdomains shifts to the opposite side of *Dxz4* in Inv-Dxz4, and almost doubles in size (Fig. 3e).

Taken together, these results demonstrate that *Dxz4* contacts a number of regions located telomeric to the locus in the WT Xi and that deletion or inversion of *Dxz4* exerts their strongest effects in the vicinity of the locus. The unidirectional nature of contacts due to the orientation of CTCF-binding sites is clearly demonstrated by contact changes observed after *Dxz4* inversion.

### TAD structure of the *Dxz4*-edited Xi

**Fig. 4:**
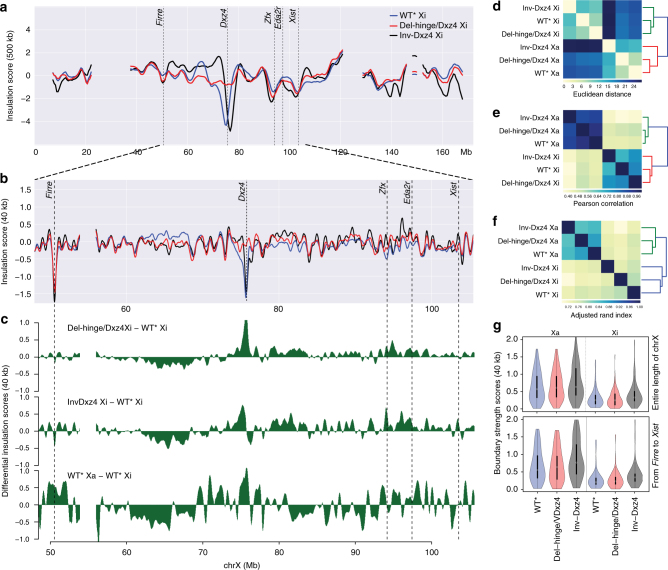
Changes in Xi TAD configuration after *Dxz4* deletion or inversion. **a** Insulation score profiles at 500 kb resolution for the whole Xi in WT* (blue), Del-hinge/Dxz4 (red), and Inv-Dxz4 (black). The positions of *Firre*, *Dxz4*, *Zfx*, *Eda2r*, and *Xist* are indicated. See Supplementary Fig. 4e, f for analysis of insulation scores of Xi and Xa in all cell lines. **b** As in **a** but based on 40 kb resolution data for a region from *Firre* to *Xist* along the X chromosome. **c** Differential insulation score profiles for Del-hinge/Dxz4 Xi (top), Inv-Dxz4 Xi (middle), and WT* Xa (bottom) relative to WT* Xi based on 40 kb resolution data for a region from *Firre* to *Xist*. **d**, **e** Hierarchical clustering based on the Euclidean distance (**d**) and Pearson correlation (using 1 − *r* as the distance measure; **e**) between standardized insulation scores at 40 kb resolution considering the Xi and Xa in WT*, Del-hinge/Dxz4, and Inv-Dxz4. See Supplementary Fig. 4g for analysis of all cell lines. **f** Hierarchical clustering based on the adjusted Rand index to quantify the correspondence between TADs called using insulation scores at 40 kb resolution along the Xi and Xa in WT*, Del-hinge/Dxz4, and Inv-Dxz4. **g** Violin plots showing the distributions of TAD boundary strength scores (see Methods) for the Xa (left) and Xi (right) in WT* (blue), Del-hinge/Dxz4 (pink), and Inv-Dxz4 (gray) for the entire X chromosome (top) and for the region from *Firre* to *Xist* (bottom). The boxes demarcate the interquartile range (IQR) with median. Whiskers are ±1.5 times the IQR

We and others reported that the mouse Xi does not display prominent TADs, in contrast to the Xa or the autosomes^7,9,10,19^. To quantify TAD number and distribution, we determined insulation scores^31^ at 500 and 40 kb resolution (Fig. 4a–c). Overall, a similar number of TADs were identified on the Xi and Xa in WT*, but insulation scores were attenuated along the Xi (Supplementary Table 2; Supplementary Fig. 4e, f). Insulation scores for Del-hinge/Dxz4 show the loss of the deep trough seen in WT* at *Dxz4* resulting in a steep peak seen in the differential insulation scores plot (Fig. 4a–c; Supplementary Fig. 4e). Inversion of *Dxz4* also results in a dramatic change in insulation scores immediately around the locus, with a rapid switch in the differential plot corresponding with a shift of the trough towards the telomere, similar to that seen in coverage score profiles (Figs. 3e and 4a–c; Supplementary Fig. 4e, f). Xi insulation scores cluster away from Xa scores (Fig. 4d, e), and a similar allelic separation is seen when comparing the extent of TAD overlap (Fig. 4f). Additionally, boundary scores and insulation score amplitudes are much greater along the Xa than the Xi, both chromosome-wide and within the *Firre*-*Xist* region (Fig. 4g; Supplementary Fig. 4f). Clustering of insulation scores suggests subtle differences in the TAD structure of Xi’s with a disrupted bipartite structure versus other clones (Supplementary Fig. 4g).

**Fig. 5:**
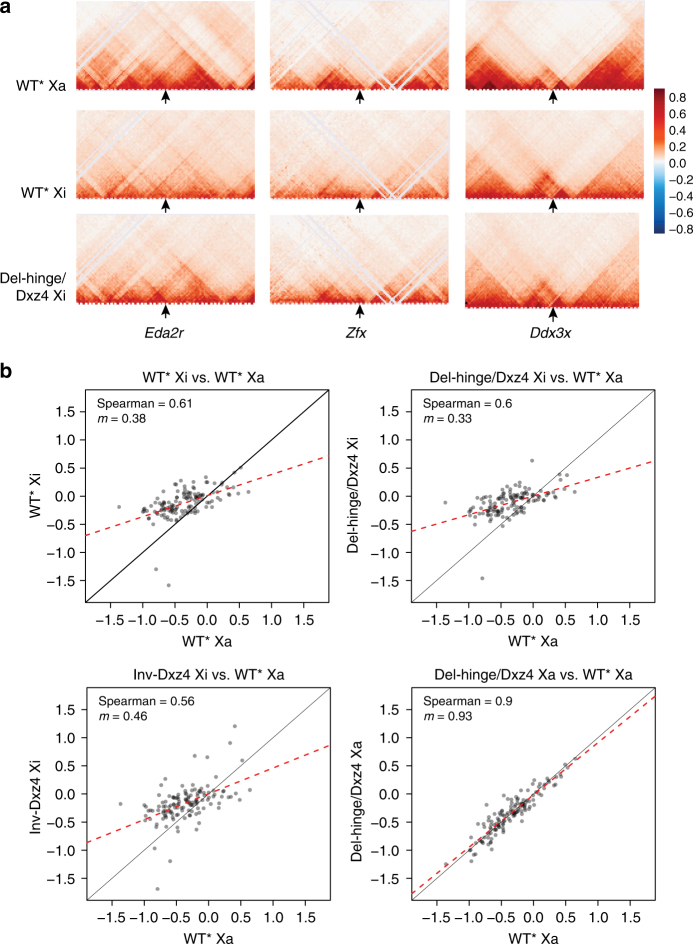
Local changes in TAD structure after *Dxz4* deletion or inversion. **a** Comparisons of contacts on the Xa and Xi in WT* and Del-hinge/Dxz4 at individual loci. Changes in TAD configuration at 40 kb resolution are shown within 4 Mb regions, each centered at a specific gene, including two genes normally subject to XCI, *Edar2* and *Zfx*, and a gene that escapes XCI, *Ddx3x*. **b** Scatter plots of WT* Xi, Del-hinge/Dxz4 Xi, Inv-Dxz4 Xi, and Del-hinge/Dxz4 Xa insulation scores at the TAD boundaries identified on WT* Xa. The dash red line shows the correlation between the samples as a linear fit, while the black line shows the expected fit for perfectly correlated data

Both Del-hinge/Dxz4 and Inv-Dxz4 show some increase in short to medium range contacts in a region 5–10 Mb telomeric to *Dxz4*, consistent with local and partial restoration of TADs (Fig. 4b, c; Supplementary Fig. 4f). Zooming in to assess potential changes in short-range contacts for specific loci normally subject to XCI (e.g., *Eda2r* and *Zfx*) shows that TADs, normally attenuated on the Xi, are partially restored in Del-hinge/Dxz4 (Fig. 5a). In contrast, loci that escape XCI, such as *Ddx3x*, show little change. A particularly large decrease in Del-hinge/Dxz4 and Inv-Dxz4 insulation scores occurs in a region between 60 and 68 Mb along the Xi, a region where WT* Xa insulation scores are also lower than WT* Xi (Fig. 4b, c; Supplementary Fig. 4f). Scatter plots of insulation scores at TAD boundary regions defined on the WT* Xa (using 40 kb bins) all show weak correlations for WT*, Del-hinge/Dxz4, and Inv-Dxz4 Xi’s versus WT* Xa, whereas control plots for the Xa’s in all three cell types show a much closer correlation, indicating that the TAD structure of the Xi is not widely restored in *Dxz4* mutants (Fig. 5b).

We conclude that the TAD structure remains largely unchanged along the Xi following *Dxz4* ablation or inversion, except around the locus itself and a few other select loci along the Xi.

### Allelic CTCF binding in *Dxz4*-edited cells

**Fig. 6:**
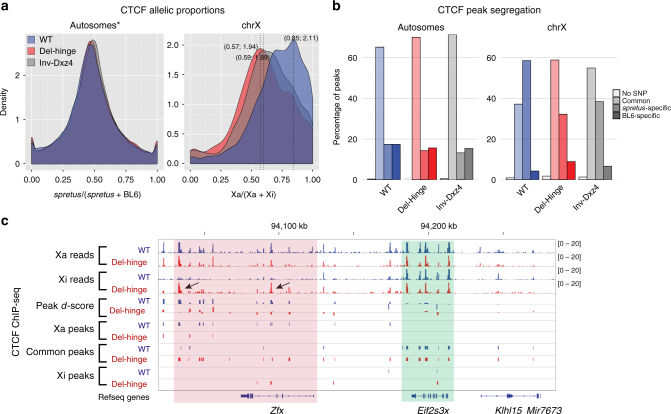
CTCF peak distribution on the Xi in Del-hinge and Inv-Dxz4. **a** Density histograms of the distribution of allelic proportions of CTCF peaks (*spretus*/(*spretus* + BL6)) along autosomes and the X chromosomes for WT (blue), Del-hinge (red), and Inv-Dxz4 (gray). The modes of the X-chromosome distributions are given in parentheses. *Chromosomes 3 and 4 were removed from the autosomes because they show aneuploidy in WT (Supplementary Fig 6). A shift in the distribution of allelic proportions due to an increase in CTCF binding on the Xi is evident for the X chromosome in Del-hinge and to a lesser extent Inv-Dxz4, compared to WT. **b** Percentages of CTCF peaks in WT (blue), Del-hinge (red), and Inv-Dxz4 along the autosomes and the X chromosomes classified as *spretus*-specific, BL6-specific, or common peaks. **c** Genome browser tracks of allelic CTCF ChIP-seq reads on the Xa and Xi, of CTCF peak *d*-scores ((Xa/(Xa + Xi) − 0.5), and of CTCF peaks assigned as Xa-specific, common, or Xi-specific for WT (blue), Del-hinge (red), and Inv-Dxz4 along a region of the X chromosome (pink background) that includes *Zfx* (a gene subject to XCI in WT, which reactivated in Del-hinge) and a region (green background) that includes *Eif2s3x* (a gene that escapes XCI). CTCF peaks that appear on the Xi in a region around *Zfx* in Del-hinge are indicated with arrows. See additional analyses in Supplementary Fig. 5–7

CTCF-binding profiles were obtained in WT, Del-hinge clone a, and Inv-Dxz4 clone a (Supplementary Fig. 5a, b; Supplementary Table 3). Average allelic proportions (see Methods) across CTCF peaks for the X chromosomes (Xa/(Xa + Xi)) were markedly different in Del-hinge (60%) and Inv-Dxz4 (63%) compared to WT (71%), but were close to the anticipated 50% for autosomes (*spretus*/(*spretus* + BL6)) in all samples (Supplementary Fig. 5c-e). Accordingly, the distribution of allelic proportions for each individual CTCF peak shows a pronounced shift toward lower values for the X chromosomes in Del-hinge, and to a lesser extent in Inv-Dxz4, compared to WT (modes of 0.57, 0.59, and 0.85, respectively; Fig. 6a; Supplementary Fig. 6). This shift cannot be attributed to a gain of Xi since ratios of BL6/*spretus* reads in input fractions of the ChIP-seq are close to one and fall within the same range as those for most autosomes in WT and Del-hinge (Supplementary Data 1). To confirm an increase in CTCF binding to the Xi seen by analyzing single-nucleotide polymorphism (SNP) coverage within diploid peaks and to further rule out an imbalance in Xi and Xa abundance between Del-hinge and WT, we called allele-specific CTCF peaks using segregated ChIP-seq reads matched to input reads. The Del-hinge/WT ratio for Xi-specific CTCF peaks called using BL6 reads (1.51) is much higher than seen for Xa-specific peaks called using *spretus* reads (1.05), or genome-wide for either BL6 or *spretus*-specific CTCF peaks (1.04 and 1.01, respectively; Supplementary Table 4). As stated above, karyotyping and FISH analyses also confirm the presence of one Xa and one Xi in a majority of cells from each cell line (see below and Methods). Analysis of Patski2-4, a WT subclone, showed an average allelic proportion of 68%, close to the 72% seen for WT (Supplementary Fig. 5e), with a distribution of allelic proportions per CTCF peak largely overlapping that of for WT (Supplementary Fig. 5f). Plots of CTCF peak *d*-scores (*spretus*/(*spretus* + BL6) − 0.5) confirm the increase in CTCF binding along the length of Xi in Del-hinge and to a lesser extent in Inv-Dxz4, whereas *d*-scores fall mainly around zero along autosomes (e.g., chromosome 2; Supplementary Fig. 7a).

Diploid CTCF peaks with sufficient SNP coverage (5×) were designated as either *spretus*-specific or BL6-specific based on an allelic proportion (*spretus*/(*spretus* + BL6)) greater than 70% or lower than 30%, respectively, with remaining peaks classified as common (Fig. 6b). The majority of X-linked CTCF peaks in Del-hinge and Inv-Dxz4 are common (59% and 55%, respectively), indicating that the increased CTCF binding on the Xi occurs at regions that bind CTCF on the Xa (Fig. 6b; Supplementary Fig. 7c). Interestingly, some of the CTCF peaks that appear on the Xi in Del-hinge occur near genes whose TAD structure and expression change. For example, at *Zfx*, a gene normally subject to XCI, new CTCF peaks (marked by arrows) located both within and downstream of the gene appear on the Xi in Del-hinge, consistent with more prominent TADs as well as reactivation (Figs. 5a and 6c; see below). In contrast, genes that escape X inactivation, for example, *Eif2s3x*, show no change in CTCF peaks (Fig. 6c).

We conclude that deletion of the hinge, and to a lesser extent inversion of *Dxz4*, results in a general increase in CTCF binding along the Xi. However, we cannot exclude that changes in CTCF binding may vary between cell clones.

### Allelic chromatin accessibility in *Dxz4*-edited cells

**Fig. 7:**
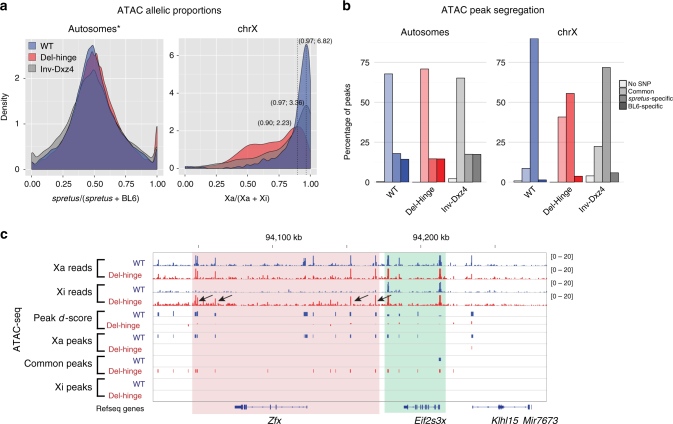
ATAC peak distribution on the Xi in Del-hinge and Inv-Dxz4. **a** As in Fig. 6a but for ATAC peaks. A shift in the distribution of allelic proportions due to an increase in ATAC peaks on the Xi is evident for the X chromosome in Del-hinge and to a lesser extent Inv-Dxz4, compared to WT. **b** As in Fig. 6b but for ATAC peaks. **c** As in Fig. 6c but for ATAC peaks. Note that the location of peaks that appear around *Zfx* (arrows) in Del-hinge differ from that of the CTCF peaks. See additional analyses in Supplementary Fig. 7–9

Chromatin accessibility was measured using ATAC-seq in WT, Del-hinge clone a, and Inv-Dxz4 clone a (Supplementary Table 5). Although the overall autosomal allelic proportions (*spretus*/(*spretus* + BL6)) were as expected (50%), the X-chromosome allelic proportions (Xa/(Xa + Xi)) for Del-hinge, and to a lesser extent for Inv-Dxz4, were lower compared to WT (70% and 78% compared to 88%), possibly due of an increase in chromatin accessibility especially in Del-hinge (Supplementary Fig. 8a-c). Accordingly, the distributions of allelic proportions (see Methods) for each ATAC peak with SNP coverage are shifted to lower values for the X chromosome, but not for autosomes (Fig. 7a; Supplementary Fig. 9). Analysis of accessibility in the Patski2-4 WT subclone showed a similar Xa bias to that seen for WT, with an average allelic proportion of 92% versus 88% in WT (Supplementary Fig. 8c) and its distribution of allelic proportions being even more skewed toward the Xa than for WT (Supplementary Fig. 8d). Plots of ATAC peak *d*-scores (*spretus*/(*spretus* + BL6) − 0.5) show that the increase in chromatin accessibility is not uniform, but occurs across the entire length of the Xi in Del-hinge and to a lesser extent in Inv-Dxz4, while *d*-scores fall mainly around zero along autosomes (e.g., chromosome 2; Supplementary Fig. 7b).

The same approach as described above for CTCF was used to designate *spretus*-specific, BL6-specific, and common peaks (Fig. 7b;). A smaller proportion of ATAC peaks are Xa-specific in Del-hinge (55%) versus WT (90%), with a higher proportion of common peaks in Del-hinge (40%) versus WT (9%; Fig. 7b). In Inv-Dxz4 these proportions are intermediate, with 72% peaks being Xa-specific and 23% common. Zooming in on individual genes, the appearance of ATAC peaks around X-linked genes that are normally inactivated and become expressed from the Xi in Del-hinge cells, for example *Zfx*, is evident, while ATAC peaks are not altered around genes that escape XCI such as *Eif2s3x* (Fig. 7c).

As seen for CTCF peaks, the additional Del-hinge and Inv-Dxz4 common ATAC peaks are found on the Xi in regions where WT common and Xa-specific ATAC peaks are also located (Supplementary Fig. 7d). Furthermore, binned CTCF and ATAC common peaks correlate both in Del-hinge (Spearman *ρ* = 0.84) and Inv-Dxz4 (*ρ* = 0.6), indicating that these two features are correlated. Indeed, unbinned common CTCF and ATAC peaks show spatial colocalization by the relative distance metric^32^ along both the Xa and the Xi in WT, Del-hinge, and Inv-Dxz4 (Supplementary Fig. 8e).

Taken together, results of the ATAC-seq analysis show an increase in Xi chromatin accessibility after deletion of the hinge and a lesser increase after inversion of *Dxz4*, consistent with a role for *Dxz4* in maintaining condensation of the Xi. Again, since we examined single-mutant clones, we cannot exclude clonal variability as an explanation to our findings.

### Epigenetic features of the *Dxz4*-edited Xi

**Fig. 8:**
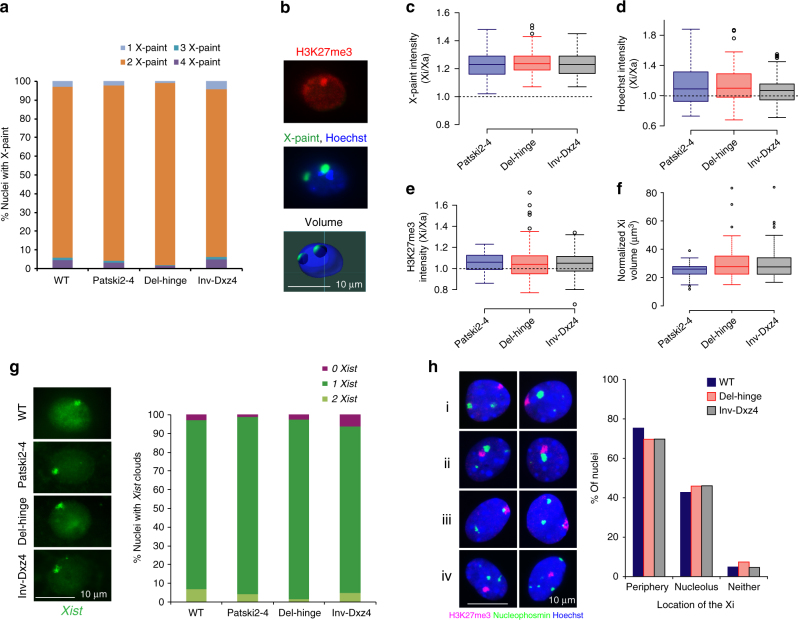
Epigenetic features of the Xi in *Dxz4*-edited cell lines. **a** Percentage of nuclei (`200 nuclei scored in each line) with 1 (light blue), 2 (orange), 3 (dark blue), or 4 (purple) X signals after DNA-FISH using an X-paint in WT, Patski2-4, Del-hinge, and Inv-Dxz4 cells. **b** Example of a *z*-stack image of a Del-hinge nucleus after H3K27me3 immunostaining (red), DNA-FISH using an X-paint (green), and Hoechst 33342 staining (blue). The Xa and Xi volumes were called based on the X-paint signals (lines show the microscope grid), using H3K27me immunostaining to identify the Xi. A 10 µm scale is shown. **c**–**e** Box plots of fluorescence intensities for the X-paint, Hoechst 33342, and H3K27me3 in wild-type Patski2-4 (blue), Del-hinge (red), and Inv-Dxz4 (gray) cells. No difference was detected between these lines by Wilcoxon rank sum test. Note that the Xi shows higher intensity of staining compared to the Xa. The boxes demarcate the interquartile range (IQR) with median. Whiskers are ±1.5 times the IQR. Outliers plotted as individual points. **f** Box plots of the Xi volume normalized to the Xa volume show a slight but significant (by Wilcoxon rank sum test) increase of the median value in the Xi volume in Del-hinge (7%; *p*-value = 0.01) and in Inv-Dxz4 (6%; *p*-value = 0.03). Boxes as described in **c**–**e**. **g** Left, examples of nuclei from WT, Patski2-4, Del-hinge, and Inv-Dxz4 cells after RNA-FISH for *Xist* (green; 10 µm scale shown). Right, bar plots of the percentage of nuclei (`200 nuclei scored in each line) with 0 (magenta), 1 (green), or 2 (yellow/green) *Xist* RNA clouds. The majority of nuclei have one *Xist* cloud with no significant difference between cell lines by Fisher’s exact test. See Supplementary Fig. 10a, b for *Xist* expression analyses. **h** Left, examples of nuclei stained with Hoechst 33342 (blue) and immunostained for H3K27me3 (red) to locate the Xi, and for nucleophosmin (green) to locate the nucleolus, show the Xi located either at the periphery (i), near the nucleolus (ii), sandwiched between periphery and nucleolus (iii), or at neither of these locations (iv). A 10 µm scale is shown. Right, the percentage of nuclei with the Xi near the periphery, the nucleolus, and at neither of these locations does not significantly differ between WT (blue), Del-hinge (red), and Inv-Dxz4 (gray) by Fisher’s exact test. A total of ~200 nuclei were scored per cell type by at least two different observers

To test for changes in the 3D volume of the Xi in *Dxz4*-edited cell lines, we used DNA-FISH for a whole X-chromosome paint. The majority of nuclei had two signals for the X-paint, consistent with the presence of two X chromosomes (Fig. 8a). H3K27me3 immunostaining resulted in a strong Xi-staining cluster in 90% of nuclei from Patski2-4, Del-hinge, and Inv-Dxz4 cells (Fig. 8b). These results confirm the presence on an Xa and Xi in these cell lines and also suggest that *Dxz4* deletion or inversion does not cause major changes in enrichment of the repressive histone mark on the Xi. The relative Xi/Xa signal intensities for the X-paint, H3K27me3 immunostaining, and Hoechst 33352 staining were comparable between cell types (Fig. 8c–e). However, the relative Xi/Xa volume determined by image analyses showed a small increase in Xi volume in Del-hinge (7%; *p*-value = 0.01 using Wilcoxon sum rank test) and in Inv-Dxz4 (6%; *p*-value = 0.03) nuclei compared to WT Patski2-4 nuclei (Fig. 8f).

RNA-FISH for *Xist* showed that the majority of nuclei had one cloud in WT (90%), Patski2-4 (95%), Del-hinge (96%), and Inv-Dxz4 (89%) cells, indicating that *Dxz4* deletion or inversion does not appear to disrupt *Xist* RNA coating on the Xi (Fig. 8g). Consistently, *Xist* expression levels were similar between cell types as shown both by RNA-seq and quantitative reverse transcription-PCR (RT-PCR; Supplementary Fig. 10a, b). We have previously reported that *Dxz4* and *Firre* on the Xi, but not on the Xa, are preferentially located near the edge of the nucleolus, and that the frequency of this preferred location and the level of H3K27me3 on the Xi decrease after knockdown of *Firre* lncRNA^15^. In contrast, we found that *Dxz4* deletion or inversion did not affect Xi positioning or H3K27me3 enrichment, as determined by nucleophosmin and H3K27me3 immunostaining (Fig. 8h).

Taken together, our results show that the changes in the configuration of the Xi we detect by Hi-C in Del-hinge/Dxz4 and Inv-Dxz4 are associated with a small increase in the apparent size of the Xi, but not with any apparent changes in H3K27me3 accumulation, *Xist* RNA coating, or location of the Xi. We cannot exclude changes that may be visible by super-resolution microscopy.

### Allelic gene expression in *Dxz4*-edited cells

**Fig. 9:**
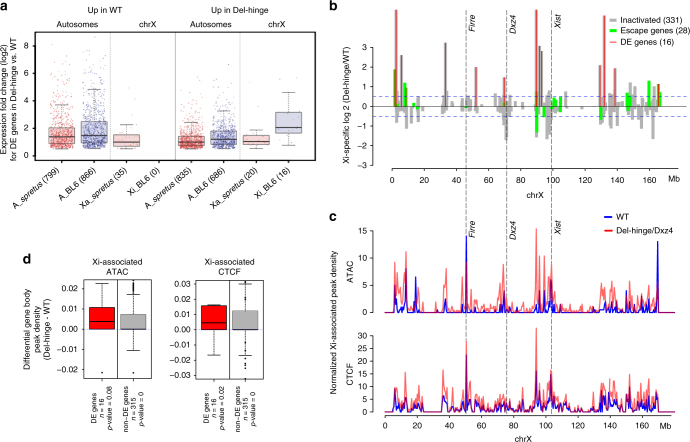
Gene expression analyses. **a** Box plots showing the distribution of fold changes in allelic gene expression in Del-hinge versus WT for autosomal (A_*spretus* or A_BL6) and X-linked (Xa or Xi) genes that have significantly increased expression (log2 > 0.5 and adjusted *p*-value < 0.05 by the Wald test) in WT or in Del-hinge as determine by DESeq2^59^. Red, *spretus* alleles; blue, BL6 alleles. Box and whisker demarcations as described for Fig. 8c–f. **b** Distribution of genes with expression fold changes between Del-hinge versus WT along the length of the Xi, considering 331 genes normally subject to XCI (gray) and 28 genes that escape XCI (green) (note only 28/29 escape genes could be tested for differential expression because *Slc16a2* lacked the allelic coverage to do so). The 16 genes that show significant differential expression are demarcated with red vertical lines. The positions of *Firre*, *Dxz4*, and *Xist* are indicated. **c** Plots of Xi-associated (common + Xi-specific) ATAC and CTCF peak density (counts binned within 500 kb windows) along the X chromosome for WT (blue) and Del-hinge (red) aligned with expression fold changes in **a**. To account for differences in the number of SNP-covered peaks obtained between samples due to differences in the depth of sequencing (Supplementary Tables 3 and 5), the binned counts are scaled by a factor obtained from the between-sample ratios of autosomal diploid SNP-covered peaks. For CTCF the scaling factors are 0.7 and 0.87, while for the ATAC peaks the scaling factors are 0.5 and 0.38, for WT and Del-hinge, respectively. The positions of *Firre*, *Dxz4*, and *Xist* are indicated. See also Supplementary Fig. 7c, d and 10c. **d** Box plots of differential Xi-associated ATAC and CTCF peak densities between Del-hinge and WT. Peak densities within X-linked gene promoter regions and gene bodies (10 kb upstream to TTS) are normalized for gene length and scaled by total diploid autosomal peak ratios. Genes are partitioned into those that show significant differential expression between WT and Del-hinge (DE; red) and those that did not (non-DE; gray). The number of genes in each group and the *p*-values from one-sided paired Wilcoxon tests are indicated. Box and whisker demarcations as described in **a**

RNA-seq was done on the edited cell lines and pair-wise comparisons were used to evaluate allelic gene expression changes (Supplementary Tables 6 and 7). In comparison to WT, Del-hinge clone a showed reactivation of a few genes on the Xi, with 16 genes showing significant upregulation, and no gene showing downregulation (Fig. 9a; Supplementary Fig. 10c; Supplementary Data 2). This unidirectional trend was not observed for the Xa, nor for the autosomes, for which 55 and 2689 genes, respectively, showed differential expression (DE) occurring in both directions (Fig. 9a; Supplementary Fig. 10c; Supplementary Data 2, 3). Note that 517 DE autosomal genes map to chromosomes 3 and 4, reflecting aneuploidy in WT cells. While a greater number (but lower proportion) of genes subject to XCI (12/331 or 4%) than escape genes (4/29 or 14%) were DE in Del-hinge clone a, the significance of this observation is unclear, given the limited number of genes classified as escape genes (Supplementary Data 4). Reactivated genes were distributed all along the Xi in regions of high gene density, with no correlation with respect to *Dxz4* location (Fig. 9b). Intriguingly, two reactivated genes (*Zfx* and *Eda2r*) show a surrounding TAD structure that becomes more similar to that of the Xa in Del-hinge clone a (Fig. 5a; Supplementary Data 2), and *Zfx* also shows an apparent increase in ATAC accessibility and CTCF binding around the gene (Figs. 6c and 7c)

Next, we examined the possibility of a correlation between increased Xi expression and increased accessibility and CTCF binding in general. The 16 reactivated genes on the Xi fall within regions with a higher density of Xi-associated ATAC and CTCF peaks (Xi-specific and common peaks binned within 500 kb) in Del-hinge clone a, suggesting that de-condensation of the Xi may facilitate limited reactivation (Fig. 9b, c; Supplementary Fig. 7c, d). We then calculated the ATAC and CTCF peak densities within all X-linked gene bodies and their upstream promoter regions (10 kb upstream of the transcription start site (TSS)). Both peak densities were greater in Del-hinge clone a for the 16 DE genes, though this trend was only significant for CTCF binding and not for accessibility (Fig. 9d). However, non-DE genes showed similar trends, indicating that although ATAC and CTCF densities along the Xi are strongly correlated (Spearman *ρ* = 0.71), they do not correlate with the upregulation of any X-linked gene in particular but rather with gene dense regions (*ρ* = 0.41 and 0.57 for ATAC and CTCF, respectively). We conclude that changes to the chromosome structure may set up an environment that allows for limited, stochastic reactivation/upregulation.

Next, we examined Xi- and Xa-specific expression levels in additional cell lines, Del-hinge clone b, Del-Dxz4, and Del-Ds-TR (Supplementary Fig. 11a-d). As many as 77–85% of genes showed no or very low Xi-specific expression (Xi-TPM < 0.2), indicating that silencing is largely maintained in these cell lines. There was a minor reactivation of the Xi in Del-hinge clone b and in Del-Dxz4, with the upregulated genes partially overlapping those observed in Del-hinge clone a, indicating variability between cell clones (Supplementary Fig. 11a-c; Supplementary Tables 6 and 7; Supplementary Data 2 and 3). In two Inv-Dxz4 clones (a and b) and corresponding subclones of WT cells (Patski2-4 a and b) there was very little evidence of DE genes on the X (8 including 4 on the Xi) and on the autosomes (317; Supplementary Fig. 10d, 11e; Supplementary Data 5). Pair-wise comparisons between cell lines confirm limited expression changes between Inv-Dxz4 and Patski2-4 (Supplementary Table 7).

To test for potential synergistic effects between condensation of the Xi and DNA methylation, we treated cells with 5-aza-2′-deoxycytidine (5-aza-2dC) to induce DNA demethylation. Consistent with the inhibitory role of DNA methylation on gene expression, there was global upregulation of autosomal and X-linked genes in WT and Del-hinge cells after 5-aza-2dC treatment (Supplementary Fig. 12a-d). In WT cells, 2328 autosomal and 129 X-linked genes were significantly upregulated, with only 640 autosomal and 27 X-linked genes being downregulated (Supplementary Fig. 12a, c). Similarly, in Del-hinge cells, 1318 autosomal and 75 X-linked genes were significantly upregulated and only 133 autosomal genes and no X-linked gene downregulated. Thus, demethylation did not cause a differential increase in the number of reactivated X-linked genes between Del-hinge and WT (Supplementary Fig. 12b, d; Supplementary Table 7; Supplementary Data 6 and 7).

Thus, deletion of *Dxz4* or of the hinge, with or without DNA demethylation, and inversion of *Dxz4* are insufficient to cause massive reactivation of genes on the Xi, despite the observed de-condensation and increase in chromatin accessibility in the edited cell lines.

## Discussion

We determined that *Dxz4* is the sole element necessary for maintenance of the condensed 3D bipartite configuration of the Xi in mouse fibroblasts. Indeed, a deletion of *Dxz4* alone (44 kb), which is much smaller than those previously reported in mouse embryonic stem (ES) cells and in human fibroblasts (200–300 kb), is sufficient to cause de-condensation of the Xi^8,9^. Remarkably, we show that the Xi bipartite structure persists after inversion of *Dxz4*, indicating that the locus insulates the Xi superdomains in either orientation. However, *Dxz4* inversion causes a massive re-distribution of long-range contacts from the telomeric to centromeric superdomain, presumably due to a reversal of the mostly unidirectional CTCF-binding motifs at *Dxz4*^12^.

The greatest level of disruption of the Xi bipartite structure after *Dxz4* deletion or inversion is in the vicinity of the locus, in agreement with a study in human where a large hinge deletion coincides with the disruption of an interaction compartment (as reflected by PC scores) in the vicinity of *DXZ4*^8^. We observed minor changes in the TAD structure of the Xi, also mostly located in the vicinity of *Dxz4*. However, the surprising increases in CTCF binding and in chromatin accessibility in cells with a deletion of the hinge, and to a lesser extent in cells with an inversion of *Dxz4*, were widespread along the Xi. CTCF binding is normally low on the Xi except at genes that escape XCI^33^, and Minajigi et al.^10^ proposed that *Xist* RNA evicts CTCF and cohesin. Furthermore, induction of *Xist* expression in male ES cells results in the formation of a bipartite structure and loss of chromatin accessibility^9^. However, our results suggest that partial CTCF return after deletion of the hinge is independent of *Xist* in our system. The increase in CTCF binding concomitant with the loss of long-range interactions on the Del-hinge Xi is reminiscent of observations made on neuronal cells with altered levels of CTCF^34^. One possibility is that loss of long-range contacts and increased chromatin accessibility may facilitate CTCF binding in cells with an alteration of the hinge.

Very little reactivation of X-linked genes, even in the presence of a demethylating agent, was observed in mutant cells, attesting to the multiple layers of control that ensure silencing of the X^1,35,36^. Rather, our findings of clonal variability suggest that de-condensation of the Xi in Del-hinge may facilitate stochastic dysregulation of a few X-linked genes (Fig. 9). A similar level of clonal variability was reported in human cells, as well as in mouse ES cells, the latter with a deletion of the hinge induced prior to XCI, in which loss of escape from XCI was observed for a single-cell clone, but was not confirmed in others^8,9^. Taken together, these results indicate that *Dxz4* mutations induced either prior to XCI or following XCI do not result in widespread changes in gene expression. No changes in the repressive histone mark H3K27me3 enrichment on the Xi were observed in our mutant lines, but we cannot exclude local changes around the locus, or a shift in Xi replication timing from early to late as observed in human cells^8^.

Since the inversion of *Dxz4*, which includes its promoter, causes massive contact re-distribution, it appears that the locus itself rather than its lncRNA is critical for the proper orientation of contacts. *Dxz4* transcription could still play a role in the Xi structure, e.g., by opening the locus, but this remains to be clarified. *Dxz4* lncRNA is differentially expressed from Xa and Xi alleles, with a long sense-transcript detected from the Xa^12^, and higher expression in female versus male tissues and cell lines consistent with Xi expression^15^.

**Fig. 10:**
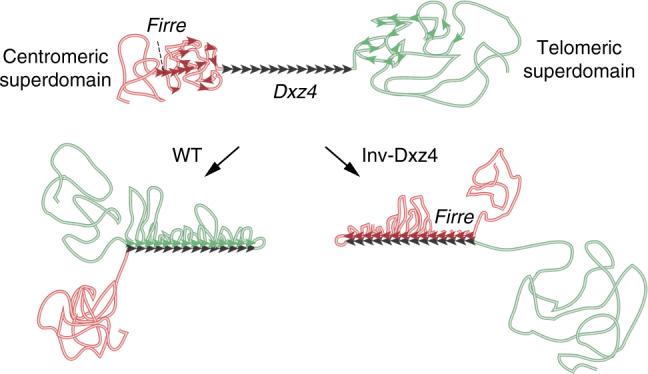
Model for the role of *Dxz4* in unidirectional contacts with other loci on the Xi. Diagram of the *Dxz4* locus (black) with adjacent centromeric (red) and telomeric (green) superdomains of the Xi. The orientation of CTCF-binding motifs is shown with black arrows, with 14 motifs being represented. Potential CTCF sites located in the telomeric superdomain are shown as green arrows, and on the centromeric domain as red arrows. In WT cells contacts between *Dxz4* and loci telomeric to the locus would result in the formation of loops anchored at *Dxz4* by the correct alignment of CTCF motifs, which would stall cohesin rings that continuously extrude loops (not depicted). *Dxz4* would be pulled to the telomeric end of the hinge. After *Dxz4* inversion contacts would shift to the centromeric superdomain, and be especially enhanced between *Dxz4* and *Firre*. A new de-condensed hinge would form, with *Dxz4* located at its centromeric end. Note that in WT and Inv-Dxz4, loops are depicted as anchored at each CTCF-binding site on *Dxz4*. However, the process of loop formation is not static, but rather highly dynamic; thus, at a given time not all loops are likely to be anchored and larger or smaller loops may form.

A surprising finding in our study is that *Dxz4* mainly contacts loci located in the telomeric superdomain and that these long-range contacts are either lost after deletion or shifted to the centromeric superdomain after inversion (Fig. 3). CTCF strongly binds at *Dxz4* on the Xi via unidirectional conserved CTCF-binding motifs embedded in the tandem repeats of the macrosatellite locus^12,15^. The orientation of CTCF motifs is critical in the formation of chromatin loops by extrusion, in which a cohesin ring complex is loaded onto two *cis* regions of chromatin, potentially in combination with other extruding factors (EFs), and then slides over the chromatin fiber in opposing directions bringing increasingly remote regions together and resulting in extrusion of a loop^11,21–23,37^. The processivity of EFs is curtailed by BEs, such as CTCF, in a directional manner, with binding to convergent motifs being far more effective^11,21–23,37^. In this scenario, the particularly large bank of Xi-specific CTCF binding at *Dxz4*^12,15^ would present a formidable barrier to EFs, halting chromatin fiber translocation at that end of the loop. Thus, EFs would tend to stall at *Dxz4*, impeded from moving through to the other side of the locus and effectively insulating the two superdomains from one another. At the other end of the loop, however, EFs would remain relatively free to continue to extrude chromatin, unless they encountered another loop or another CTCF-binding site preferably in opposite orientation (Fig. 10). Given the paucity of CTCF binding on the Xi this would result in very large loops spooling out from the *Dxz4* locus bridging relatively few contact hotspots. This is consistent with polymer simulations highlighting the role of EFs in imposing insulation over large spatial and genomic distances simply by their translocation processivity being regulated by BEs such as CTCF^21^. Following this reasoning, regions immediately to the rear of *Dxz4* (with directionality dictated by the orientation of the CTCF motifs) would be subject to reduced translocation, due to stalling of the EFs. The diminished extrusion would result in fewer long-range contacts and reduced loop formation with chromatin taking on a more unraveled appearance (Fig. 10). This unraveling “behind” *Dxz4* may also result from increased tension in the chromatin fiber due to the locus being pulled closer to *Xist* and *Firre* in the WT and Inv-Dxz4 Xi, respectively (Fig. 3e). The *Dxz4* orientation-dependent shift in contacts is also visible as a telomeric to centromeric switch in the line of strong contacts made by *Dxz4*, representing a “flame”^21,29^, in line with the model proposed above (Figs. 1a, 2b, and 3b).

Among the new or enhanced long-range contacts found after *Dxz4* inversion, the most striking is a strong interaction between *Dxz4* and *Firre*, most likely mediated by the banks of CTCF-binding sites at both loci^12,15,28^. While the orientation of CTCF motifs is not completely defined at *Firre* due to the repetitive nature of the locus, our motif searches indicate that a similar number of CTCF-binding sites exist in either orientation. Note that a previous study reported a strong peak of *DXZ4/FIRRE* interactions in human cells, while we observed little interaction in mouse WT cells^8,11^. This probably reflects a different configuration between species, with the human *DXZ4* locus containing two sets of CTCF motifs in opposite orientation^14^, which would be favorable to interactions with loci on both sides, including *FIRRE*. In human *DXZ4* colocalizes with a strong visible accumulation of CTCF^38,39^. We found that the bank of CTCF-binding sites at *Dxz4* still has the ability to curtail the movement of cohesin even when inverted (Fig. 10). Thus, *Dxz4* contacts have little specificity, consistent with the lack of conservation of the *Dxz4* repeat units, except for the CTCF-binding motifs and a short sequence (13 bp) that may represent a binding site for another BE^12^. A previous study reported that cohesin is excluded from the Xi^10^, but we have reported that at least *Dxz4* and *Firre* strongly bind cohesin on the Xi^15^. Further studies are needed to sort out the role of cohesin and CTCF on the Xi. Indeed, loss of CTCF can lead to re-distribution of cohesin away from CTCF binding peaks rather than a loss of cohesin^40^.

We propose a model in which the multiple CTCF sites within *Dxz4* act as a row of unidirectional obstacles that hook multiple loops of chromatin with an inherent ability to insulate in either orientation (Fig. 10). This model also suggests a possible mechanism by which the Xi may fold at the hinge, but this remains to be further investigated^6^. The *Dxz4* platform may behave in a manner more akin to a ratchet than to velcro, since the locus orientation is important and translocation of chromatin is impeded unidirectionally. Although Xi-specific *Dxz4* contacts are represented as occurring simultaneously along the same chromatin fiber in our model and could be interpreted to be stable structures, in reality this is unlikely to be the case. The process is probably dynamic, with contacts between distant X-linked loci and the *Dxz4* platform being transient albeit relatively frequent. And whether some or all units of the Xi tandem repeat are engaged in loop formation in a single cell is unclear. Single-cell analyses may help better understand cell-to-cell variability in the Xi contact distribution.

## Methods

### Allele-specific CRISPR/Cas9 editing of Patski cells

Patski cells are fibroblasts that we previously derived from 18dpc embryonic kidney from a cross between a BL6 female mouse with an *Hprt*^*BM3*^ mutation^41^ and *Mus spretus* male. Frequent polymorphisms between mouse species (1/93 bp using validated Sanger *M. spretus* SNPs compared to mm10) allow distinction between alleles. The cells were selected in HAT media such that the BL6 X chromosome is always inactive as verified in previous studies^26,27^. Patski cells were cultured in Dulbecco’s modified Eagle medium with 10% fetal bovine serum and 1% penicillin/streptomycin. Xi-specific deletions or inversions were induced using pairs of small guide RNAs (sgRNAs) that flank either most of the hinge region or only some of its elements (Supplementary Table 8). The allele-specific sgRNAs designed using CHOPCHOP^42,43^ were selected to include BL6 SNPs at the PAM site if available (Supplementary Fig. 1a; Supplementary Table 8). Patski cells were transfected using Ultracruz transfection reagents (Santa Cruz). Verifications of the deletions/inversion were done using PCR together with Sanger sequencing to confirm specific loss of the BL6 allele at the deletion and to verify junction sequences containing BL6 SNPs (Supplementary Fig. 1b; Supplementary Table 9). Note that two independent clones (a and b) with a deletion of the hinge and with an inversion of *Dxz4* were derived, while single clones were derived for other deletions or inversion. Del-hinge clone a was also confirmed by FISH (Supplementary Fig. 1c). The presence of normal un-rearranged X chromosomes was verified by karyotyping. Del-hinge clone a and Inv-Dxz4 clone a, which have a near-diploid normal karyotype, were used for all studies (Hi-C, ChIP-seq, ATAC-seq, and microscopy), except for gene expression analyses in which we considered both clones a and b. The main WT Patski cell line is near-diploid, with 40,XX cells as well as cells with trisomy 3 and monosomy 4. A WT 40,XX subclone of WT Patski, Patski2-4, was also derived. Genomic DNA sequencing verified the presence of similar number of reads for the X chromosomes from BL6 (Xi) and from *spretus* (Xa) in WT cells with an allelic ratio similar to that seen for autosomes except for chromosomes 3 and 4, which show skewing due to known BL6 trisomy 3 and monosomy 4 in a high proportion of WT cells, not present in Del-hinge and Inv-Dxz4 clones. (Supplementary Data 1).

### DNA-FISH, RNA-FISH and immunostaining

To verify the presence of a deletion in Del-hinge DNA-FISH was done on metaphase cells fixed in methanol:acetic acid (3:1 volume) using BAC probes (RP23-299L1 for *Dxz4* and RP24-322N20 for *Firre*). Probes were labeled with SpectrumGreen or SpectrumRed dUTP (Vysis # 02N32-050 or 02N34-050) using a nick translation reagent kit (Abbott Molecular Inc.). Probes were hybridized overnight at 42 °C to slides denatured in 70% formamide/2× SSC at 85 °C for 15 min and dehydrated for 2 min each in 70, 85, and 100% ethanol, followed by post-hybridization washing three times 5 min in 50% formamide/2× SSC at 42 °C and three times 5 min in 2× SSC at 42 °C. Counterstaining was done with 33342 Hoechst in phosphate-buffered saline (PBS; 2 µg/ml), prior to mounting the slides in Antifade.

RNA-FISH was done using a 10 kb *Xist* cDNA plasmid (pXho, which contains most of exon 1 of *Xist*^44^) labeled by nick translation using green dUTP (Abbott Molecular Inc., # 02N32-050). Cells were permeabilized in buffer (100 mM NaCl, 300 mM sucrose, 3 mM MgCl_2_, and 10 nM PIPES, pH 6.8) containing 0.5% Triton and an RNase inhibitor (2 nM vanadyl ribonucleoside complex) on ice for 10 min prior to fixation in 3% paraformaldehyde for 10 min at room temperature. Slides were dehydrated for 2 min each in 70, 80, 95, and 100% ethanol. Hybridization to the probe was done overnight at 37 °C prior to washing three times 5 min in 50% formamide/2× SSC at 42 °C and three times 5 min in 2× SSC at 42 °C. Slides were counterstained with 33342 Hoechst in PBS (2 µg/ml, Molecular probes). A total of ~200 nuclei per genotype were scored for the presence of *Xist* RNA clouds.

Immunostaining of paraformaldehyde-fixed nuclei was done using primary antibodies for H3K27me3 (rabbit-derived polyclonal antibody diluted 1:250, Upstate/Millipore, # 07-449) and for nucleophosmin (mouse-derived monoclonal antibody diluted 1:250, Abcam, # ab10530). Briefly, cells were cultured on chamber slides and permeabilized in 0.5% Triton X-100 for 10 min prior to fixation with 4% paraformaldehyde for 10 min. Fixed cells were blocked in bovine serum albumin (BSA) buffer (4× SSC and 0.42% BSA) for 30 min prior to incubation with the primary antibody overnight at 4 °C. Cells were then washed with PBS with 0.05% Tween and slides were incubated with secondary antibodies diluted 1:300 (Texas red anti-rabbit from Vector # TI-1000, and fluorescein anti-mouse, Vector, # FI-2100) for 2 h at 37 °C. Slides were washed in PBS with 0.05% Tween and counterstained with Hoechst 33342 (2 µg/ml, Molecular Probes). Nuclei were examined by fluorescence microscopy to score the position of the Xi marked by H3K27me3 with respect to the nuclear periphery and the edge of the nucleolus. For Xi nuclear positioning, a total of ~200 nuclei were scored by at least two different observers.

For 3D image analysis, DNA-FISH using a whole mouse X chromosome paint probe (XMP X green from MetaSystems) was done as described above in combination with H3K27me3 immunostaining and Hoechst 33342 staining (2 µg/ml, Molecular Probes). Images were collected using 21 *z*-stacks (0.3 µm per stack; 10 stacks up and down from the middle plane) using a Nikon TiE inverted wide-field fluorescence microscope. The 3D image analysis was done using Imaris image analysis software for 63, 86, and 51 nuclei from Patski2-4, Del-hinge, and Inv-Dxz4, respectively, in which the X-chromosome surface was called based on X-paint probe intensity using the auto-setting threshold. As expected, the condensed Xi marked by H3K27me3 usually showed stronger signals for the X-paint probe compared to the Xa, and also had a stronger Hoechst 33342 signal. The Xi volume was calculated and normalized to the Xa volume in the same nucleus (Xi/Xa×15 µm^3^).

### Quantitative RT-PCR

RNA extracted from WT, Patski2-4, Del-hinge, and Inv-Dxz4 cells by the Qiagen RNeasy kit with on-column DNaseI digestion was reverse-transcribed into first-strand cDNA using SuperScriptII reverse transcriptase (Invitrogen). qPCR was done for two independent cDNA preparations per genotype using gene-specific primers and an ABI7900 qPCR system. The primer sequence information is in Supplementary Table 9.

### In situ DNase Hi-C data analysis

In situ DNase Hi-C was done on intact nuclei from WT and deleted/inverted Patski cells using a previously described method^45^. We sequenced the in situ DNase Hi-C libraries using paired-end reads 80 and 150 bp in length (Supplementary Table 1). For each in situ DNase Hi-C library, we mapped each end of the paired-end reads separately to the BL6 genome using the NCBI build v38/mm10 reference genome assembly obtained from the UCSC Genome Browser^46^ and a pseudo-*spretus* genome using BWA-MEM (v0.7.3) in single-end mode using default parameters^47,48^. A pseudo-*spretus* genome was assembled by substituting available SNPs (from Sanger Institute, SNP database 2014/10/27 v4) into the BL6 reference genome, as described^33^. We retained only primary reads with mapping quality (MAPQ)  ≥ 30 for further analysis. Using heterozygous SNPs between the BL6 genome and the pseudo-*spretus* genome that were validated for our particular Patski cell line, we segregated all high-quality uniquely mapped reads (MAPQ ≥ 30) using an approach very similar to that described in ref. ^7^, with the exception that in order to maximize the number of reads assigned to either the BL6 Xi or the *spretus* Xa, we required that only one end of the read be specifically mapped to one mouse species, while the other end was allowed to be ambiguously mapped. This approach, commonly used to analyze data from hybrid systems, is based on the assumption that intrachromosomal contacts are much more frequent than interchromosomal contacts. Briefly, each end of each read pair was assigned to one of three categories: (1) BL6-SNP reads containing only BL6-specific SNP(s); (2) *spretus*-SNP reads containing only *spretus*-specific SNP(s); (3) ambiguous reads that did not contain valid SNPs or that contained valid SNPs from both alleles. We refer to both BL6-SNP reads and *spretus*-SNP reads as “allele-specific reads”, and reads that do not contain valid SNPs as “allele-uncertain reads”. Reads were paired with their corresponding mates, and those read pairs with at least one end being allele-specific were retained for subsequent allele-specific analysis. To eliminate the bias due to the PCR duplication step, we removed redundant paired-end reads defined as those pairs where both ends were mapped to identical locations in the same genome assembly. This resulted in a set of valid read pairs representing DNA–DNA interactions (Supplementary Table 1).

We generated tables of allelic read counts by chromosome and genome-wide along with the allelic ratio (BL6/*spretus*) and the ratio relative to the genome-wide ratio for Patski WT, Del-hinge, and Inv-Dxz4 in situ DNase Hi-C libraries (Supplementary Data 1). Tables were generated for (1) all read pairs (*cis* + trans), (2) interchromosomal read pairs (*trans* only), (3) intrachromosomal reads pairs (*cis* only), and (4) intrachromosomal reads pairs separated by at least 20 kb (*cis* > 20 kb). Note that in the case of (1) and (2) counts are based on each end of filtered and segregated read pairs, while for (3) and (4) counts are based on filtered and segregated read pairs. The tables show a similar number of reads for the X chromosomes from BL6 (Xi) and from *spretus* (Xa) in WT cells with an allelic ratio similar to that seen for autosomes except for chromosomes 3 and 4 in WT cells, which show skewing due to known BL6 trisomy 3 and monosomy 4 in a high proportion of WT cells, not present in Del-hinge and Inv-Dxz4 clones. Using all read pairs (*cis* + trans), the X-chromosome allelic ratio relative to the genome is slightly skewed toward the Xi (1.08, 1.2, and 1.21 for WT, Del-hinge, and Inv-Dxz4, respectively). However, this appears to be driven by intrachromosomal interactions rather than by potential copy number differences, and by using only interchromosomal read pairs (*trans* only), the X-chromosome allelic ratio relative to the genome is close to 1 in all samples (1.01, 1.01, and 1.03 for WT, Del-hinge, and Inv-Dxz4, respectively). Furthermore, no such skewing in the allelic ratios for the X chromosome are seen for WT genomic DNA, nor for WT or Del-hinge ChIP input (Supplementary Table 3).

The resulting valid read pairs were used to generate allele-specific whole-genome contact maps at 500 and 40 kb resolution. To do so, we partitioned the genome into non-overlapping bins and counted the number of allele-specific contacts (uniquely mapped valid paired-end reads) observed between each pair of bins. The dimension of the resulting allelic contact maps is the total number of bins in the genome, where entry (*i*, *j*) contains the contact count between bins *i* and *j*. We normalized the allele-specific contact maps using an iterative correction method^49^ to obtain a filtered contact map with near-equal row and column sums. Prior to applying the iterative correction procedure, the contact maps were filtered as follows: bins along the diagonal, super-diagonal (+1 off-diagonal), and sub-diagonal (−1 off-diagonal) (representing entries dominated by self-ligation products), and bins with the lowest 2% read coverage (representing sparsely populated regions dominated by spurious contacts) had their contact counts set to zero.

To increase resolution, two pooled sets of Hi-C contacts were generated: a data set representing Del-hinge and Del-Dxz4 (designated Del-hinge/Dxz4) and another representing WT and Del-Ds-TR (designated WT*; Supplementary Table 1). Allelic contact maps for each individual data set are very similar to those obtained from the pooled data in terms of the presence or absence of the bipartite structure, justifying the pooling (Fig. 1a; Supplementary Fig. 2a, b).

To compare contact maps across multiple samples, intrachromosomal contact maps were quantile normalized to one another. The Pearson correlation transformation of contact matrices was performed to better visualize the more probable contacts and mitigate for differences in sequencing depth and data sparsity due to allelic-specific read assignment. This transformation was performed as described in ref. ^6^, except that the transformation was performed on the ICE (iterative correction and eigenvector decomposition)-normalized contact matrices rather than the observed-over-expected matrices because we did not wish to normalize out the distance effect.

### Contact decay curves

Plots of the average Hi-C interaction frequencies as a function of genomic distance^6^ were performed using ICE-normalized contact matrices at 500 kb resolution either across the length of the chromosomes or within the specified regions of the X chromosome. The value in the contact decay curve at *x*-axis position *k* is the normalized sum of read counts for all intrachromosomal interactions involving pairs of loci (*i*, *j*) such that *i* − *j* = *k* or *j* − *i* = *k*. The curve is the same if we consider only the upper triangle (unidirectional) or both upper and lower triangles (bidirectional), as long as we divide through by the correct total count in each case. To account for differences that arise for technical reasons during library preparation, the average autosomal reads per unit of distance from the diagonal for each sample and allele were scale normalized to one another. These autosomal scaling factors were then applied to the X-chromosome alleles.

### Coverage score analysis

To count the number of reads spanning each locus along the chromosome across all length scales we made a minor adaptation to the coverage score developed by^30^. Briefly, the coverage score for each bin at a particular resolution was calculated as the average number of interactions within bins spanning this central bin of interest. This score calculation can be visualized as sliding a V-shaped region (with the arms of the V extending all the way to the edges of the contact map) along the diagonal of the contact map and calculating the mean interaction counts (sum of counts/number of bins) within the region. One point of difference compared to Eser et al.^30^ was that instead of normalizing the coverage score for each chromosome to fall within the range [0; 1], we normalized the scores by calculating log2(coverage score/chromosomal mean), as is done in the insulation score calculation (see below). Additionally, we did not restrict our analysis to interactions <100 kb, but instead considered all interactions spanning each locus (i.e., the arms of the V-shaped region extend all the way to the borders of the contact map). However, when calculating the coverage scores for the unmerged datasets (WT, Del-hinge, Del-Dxz4, Del-Ds-TR, and Inv-5′ Ds-TR), we did exclude bins within the first and last 10 Mb of the chromosome in order to avoid extreme edge effects that were evident especially in the sparser samples. Furthermore, a five-window, degree two polynomial Savitzky-Golay filter was applied to the resulting vector to smooth the signal, with interpolated values being assigned to edge-case bins. A standardized smoothed coverage score (coverage *z*-score) or a coverage score rescaled to lie within the [0; 1] interval was used where described in text.

### Insulation score and domain boundary analysis

To determine regions of more localized interaction (domains) and associated boundaries, we used the insulation score method introduced by Crane et al.^31^. Insulation score scripts were downloaded from https://github.com/dekkerlab/cworld-dekker. For 500 kb binned DNase Hi-C counts, insulation scores were called using the script matrix2insulation.pl with options --is 3500001 –immean --ss 1000001 --ids 2000001 --nt 0.01 --bmoe 3, while for 40 kb data the parameters used were --is 520001 --immean --ss 160001 --ids 320001 --nt 0.01 --bmoe 3. TADs were called using the script insulation2tads.pl using the default parameters (--mbs 0 --mts 0, representing minimum boundary strength and minimum TAD strength, respectively) (Supplementary Table 2). Boundary strength (Fig. 4g) is defined as the difference in the delta vector between the local maximum to the left and local minimum to the right of the boundary bin.

### CTCF ChIP-seq analysis

ChIP-seq was performed on WT and edited Patski cells using an antibody for CTCF (Upstate/Millipore, # 07-729-25UL) and an established protocol^50,51^. Briefly, chromatin was crosslinked with 1% formaldehyde chromatin, sonicated to ~500 bp fragments and immunoprecipitated overnight with the CTCF antibody followed by pull down using protein A sepharose beads (Amersham). Controls included the input fraction. DNA was purified from the ChIP and input control fractions using a PCR purification kit (Qiagen). PCR analyses confirmed loss of CTCF binding at the deleted *Dxz4* locus in Del-hinge cells, while CTCF binding at the imprinting control region near *H19* was maintained (Supplementary Fig. 5a, b; Supplementary Table 9). CTCF ChIP and input libraries were sequenced as paired-end reads of 75 bp in length (Supplementary Table 3). CTCF and input ChIP-seq paired-end reads were mapped to the BL6 genome using the NCBI build v38/mm10 reference genome assembly obtained from the UCSC Genome Browser^46^ using BWA-MEM (v0.7.3) in paired-end mode using default parameters^47,48^. Primary mapped valid paired reads with MAPQ ≥ 10 were de-duplicated and used for downstream analysis (Supplementary Table 3).

Additionally, input ChIP-seq paired-end reads were also mapped to the pseudo-*spretus* assembly described in the In situ DNase Hi-C data analysis section of the Methods (above) in order to assign reads to each parental allele where possible and compare their numbers across chromosomes (Supplementary Table 4). These data were used to generate tables of allelic read counts per chromosome and genome-wide along with the allelic ratio (BL6/*spretus*) and the ratio relative to the genome-wide ratio for both the Patski WT and Del-hinge ChIP libraries (Supplementary Data 1). The tables show that a similar number of reads for the X chromosomes from BL6 (Xi) and from *spretus* (Xa) in WT cells with an allelic ratio within the range seen for autosomes except for chromosomes 3 and 4 in WT cells, which show skewing due to known BL6 trisomy 3 and monosomy 4 in a high proportion of WT cells, not present in Del-hinge and Inv-Dxz4 clones. Furthermore, no skewing in the allelic ratios for the X chromosome is seen for WT genomic DNA, nor for WT, Del-hinge, and Inv-Dxz4 in situ DNase Hi-C libraries (Supplementary Data 1, see above).

Segregated CTCF ChIP-seq and input reads were also used to call allele-specific CTCF peaks using MACS2^52^ (Supplementary Table 4). Allelic CTCF peak numbers throughout the genome and along the X chromosome were determined for both WT and Del-hinge (Supplementary Table 4). Additionally, the BL6/*spretus* ratios was calculated for reads and peaks, as well as the Del-hinge/WT ratios of peak numbers, with Del-hinge/WT peak abundance ratios scaled by a correction factor based on the genome-wide WT/Del-hinge ratio of the total number of peaks (1.13) (Supplementary Table 4).

We also called diploid (non-allelic) CTCF peaks using all reads (unsegregated) after normalization to their chromatin inputs also using MACS2^52^ (Supplementary Table 3). Over 80% of these peaks contained a validated BL6/*spretus* SNP (Supplementary Fig. 5c), with the vast majority covered by at least five reads and considered for allelic analysis (Supplementary Fig. 5d). CTCF peaks containing *spretus*/BL6 SNPs that were covered by a total of at least five reads were considered for allelic analysis (Supplementary Fig. 5; Supplementary Table 3). This level of coverage was chosen because the overall allelic proportion (*spretus*/(*spretus* + BL6)) did not change much regardless of whether 5× or 10× coverage thresholds were used (Supplementary Fig. 5e).

For each non-allelic CTCF peak with sufficient SNP coverage, an allelic proportion of SNP read coverage (*spretus*/(*spretus* + BL6)) was calculated. For autosomes, distributions of allelic proportions fell around 0.5 indicating that peaks were common to both alleles, as expected (Fig. 6a, Supplementary Fig. 6). Chromosomes 3 and 4 in WT were anticipated exceptions due to known BL6 trisomy 3 and monosomy 4 in a high proportion of WT cells, not present in Del-hinge and Inv-Dxz4 clones. This served as useful validation, but these chromosomes were excluded from subsequent aggregate autosomal analysis (Fig. 6a; Supplementary Fig. 6). In contrast to the diploid autosomes, distributions of allelic proportions for the X chromosome were skewed toward the Xa (Fig. 6a, Supplementary Fig. 6). Small peaks at 0 (BL6-specific peaks) and 1 (*spretus*-specific peaks) in the distributions arise due to lack of coverage in some regions.

Using autosomal distributions of allelic proportion as a guide, peaks with an allelic proportion of >0.7 were designated as *spretus*/Xa-specific, while those with an allelic proportion of <0.3 were deemed to be BL6/Xi-specific. Peaks with an allelic proportion falling within the range [0.3; 0.7] were considered common to both alleles (Supplementary Fig. 5; Supplementary Table 3).

Subtracting 0.5 from allelic proportions resulted in “*d*-scores” as previously described^9,53^, with values ranging from −0.5 to +0.5. Peaks with positive *d*-scores were covered by more reads emanating from the *spretus*/Xa allele, while those peaks having negative *d*-scores showed a BL6/Xi bias. Imprinting control regions near *H19* (maternally expressed gene) and within *Peg3* (paternally expressed gene) on chromosome 7 showed CTCF peaks only on the maternal and paternal allele, respectively (Supplementary Fig. 7a).

### ATAC-seq analysis

ATAC-seq was done on WT and edited Patski cells using a published method^54^. ATAC-seq libraries were sequenced in multiple runs as paired-end reads of 75 or 150 bp in length (Supplementary Table 5). ATAC-seq read mapping, peak calling, allelic assignment, and *d*-score calculation were performed as described for CTCF peaks, except that ATAC peaks were called without using an input (background) library and all reads were trimmed to 75 bp prior to mapping for the sake of consistency (Supplementary Fig. 8; Supplementary Table 5). A smaller proportion of ATAC peaks contained SNPs than CTCF peaks (Supplementary Fig. 8a), which could be due to the fact that in both WT and Del-hinge many more, smaller ATAC peaks were called compared to CTCF peaks, possibly a consequence of not having background data to compare against as one does for ChIP-seq with input samples. However, the majority of peaks harboring a SNP were covered by at least five SNP reads (Supplementary Fig. 8b). The overall allelic proportion (*spretus*/(*spretus* + BL6)) for ATAC peaks did not change much regardless of whether 5× or 10× coverage was required (Supplementary Fig. 8c).

For each ATAC peak with sufficient SNP coverage, an allelic proportion of SNP read coverage (*spretus*/(*spretus* + BL6)) was calculated and, as described for CTCF peaks distributions of allelic proportions for autosomes fell around 0.5 indicating that peaks were common to both alleles, as expected (Fig. 7a; Supplementary Fig. 9). Once again, the exceptions to this were chromosomes 3 and 4 in WT due to known BL6 trisomy 3 and monosomy 4 in a high proportion of WT cells, not present in Del-hinge and Inv-Dxz4 clones, which acted as useful validation, with these two chromosomes excluded from subsequent aggregate autosomal analysis (Fig. 7a; Supplementary Fig. 9). In contrast to the diploid autosomes, distributions of allelic proportions for the X chromosome were skewed toward the Xa (Fig. 7a; Supplementary Fig. 9). Small peaks at 0 (BL6-specific peaks) and 1 (*spretus*-specific peaks) in the distributions arise due to lack of coverage in some regions. Imprinting control regions near *H19* (maternally expressed gene) and within *Peg3* (paternally expressed gene) on chromosome 7 showed ATAC peaks only on the maternal and paternal allele, respectively (Supplementary Fig. 7b).

### RNA-seq analysis

RNA-seq indexed libraries were prepared using Illumina TruSeq RNA sample preparation kit on WT, Del-hinge (clone a and b), Del-Dxz4, and Del-Ds-TR cells, as well as on Inv-Dxz4 (clone a and b) and the WT subclone Patski2-4 (used to derive Inv-Dxz4). By RT-PCR, *Dxz4* expression was similar in Del-hinge, Inv-Dxz4, and WT, but we could not separate expression from the Xa or Xi, due to lack of informative SNPs. To induce DNA demethylation a 48 h treatment using 4 µM 5-aza-2dC was used, followed by a 24 h recovery period, reported to lead to 2% of cells with reactivation of an X-linked green fluorescent protein reporter gene^55^. We found that 5-aza-2dC treatment caused 20% more cell death in WT and Del-hinge cells compared to the mock treatment, similar to that reported for dermal fibroblasts^55^.

RNA-seq libraries were sequenced as single-end reads of 75 bp in length (Supplementary Table 6). RNA-seq reads were mapped to the UCSC mm10 (NCBI build v38) refSeq transcriptome^46^ as downloaded and packaged in the iGenomes reference sequences and annotation files on July 17, 2015. (https://support.illumina.com/sequencing/sequencing_software/igenome.html). Tophat2 (v 2.0.12) (calling bowtie2 (v2.2.3)) was used to perform single-end mapping allowing six mismatches but otherwise default parameters^56,57^. To determine biallelic expression levels, mapped reads were assigned to refSeq genes using HT-seq^58^ and counts were converted into TPMs using custom R scripts.

Genes containing *spretus*/BL6 SNPs that were covered by a total of at least five reads were considered for allelic analysis. For each gene with sufficient SNP coverage, an allelic proportion of SNP read coverage (*spretus*/(*spretus* + BL6)) was calculated. Read counts for each gene were then distributed to each allele based on this SNP read allelic proportion, allowing us to perform DE between samples for each allele. DE analysis was performed using DESeq2^59^ (Supplementary Table 7). WT and Del-hinge control samples from the 5-aza-2dC experiment were pooled and treated as biological replicates for the WT and Del-hinge clone a samples.

Genes were deemed to escape XCI in the Patski WT line if their expression levels met the similar criteria to those used by Berletch et al.^33^ in 2/3 of the WT samples (WT and the two WT 5-aza-2dC untreated replicates; Supplementary Data 4): (1) the 99% lower confidence limit (alpha = 0.01) of the escape probability was >0.01 based on a binomial distribution parameterized by the expected proportion of reads from the Xi indicating some contribution from the Xi; (2) the diploid gene expression measured by TPM was  ≥ 1, indicating that the gene was expressed; (3) the Xi-TPM was ≥0.1, representing sufficient reads from the Xi; and (4) the SNP coverage was  ≥ 5 for each gene. A similar set of genes that escape XCI in WT cells were found as in our previous study (29 escape genes; Supplementary Data 4)^33^.

### Data availability

All sequencing data that support the findings of this study have been deposited in the National Centre for Biotechnology Information GEO and are accessible through the GEO SuperSeries GSE59779 as SubSeries GSE107282, GSE107286, GSE107290, and GSE107291. All other data and the scripts used for the analyses that support the findings of this study are available from the corresponding authors upon reasonable request.

## Electronic supplementary material

Supplementary Information

Description of Additional Supplementary Files

Supplementary Data 1

Supplementary Data 2

Supplementary Data 3

Supplementary Data 4

Supplementary Data 5

Supplementary Data 6

Supplementary Data 7
