# Supplementary material for: Orientation-dependent Dxz4 contacts shape the 3D structure of the inactive X chromosome
Source: Nat Commun. 2018 Apr 13;9:1445. doi: 10.1038/s41467-018-03694-y (PMC5899087; doi:10.1038/s41467-018-03694-y)
Supplement: Supplementary file 1 — Supplementary Information [file 41467_2018_3694_MOESM1_ESM.pdf]

Orientation-dependent *Dxz4* contacts shape the 3D structure of the inactive X chromosome

Bonora, Deng et al.

Supplementary Figures 1 – 12

Supplementary Tables 1 – 9

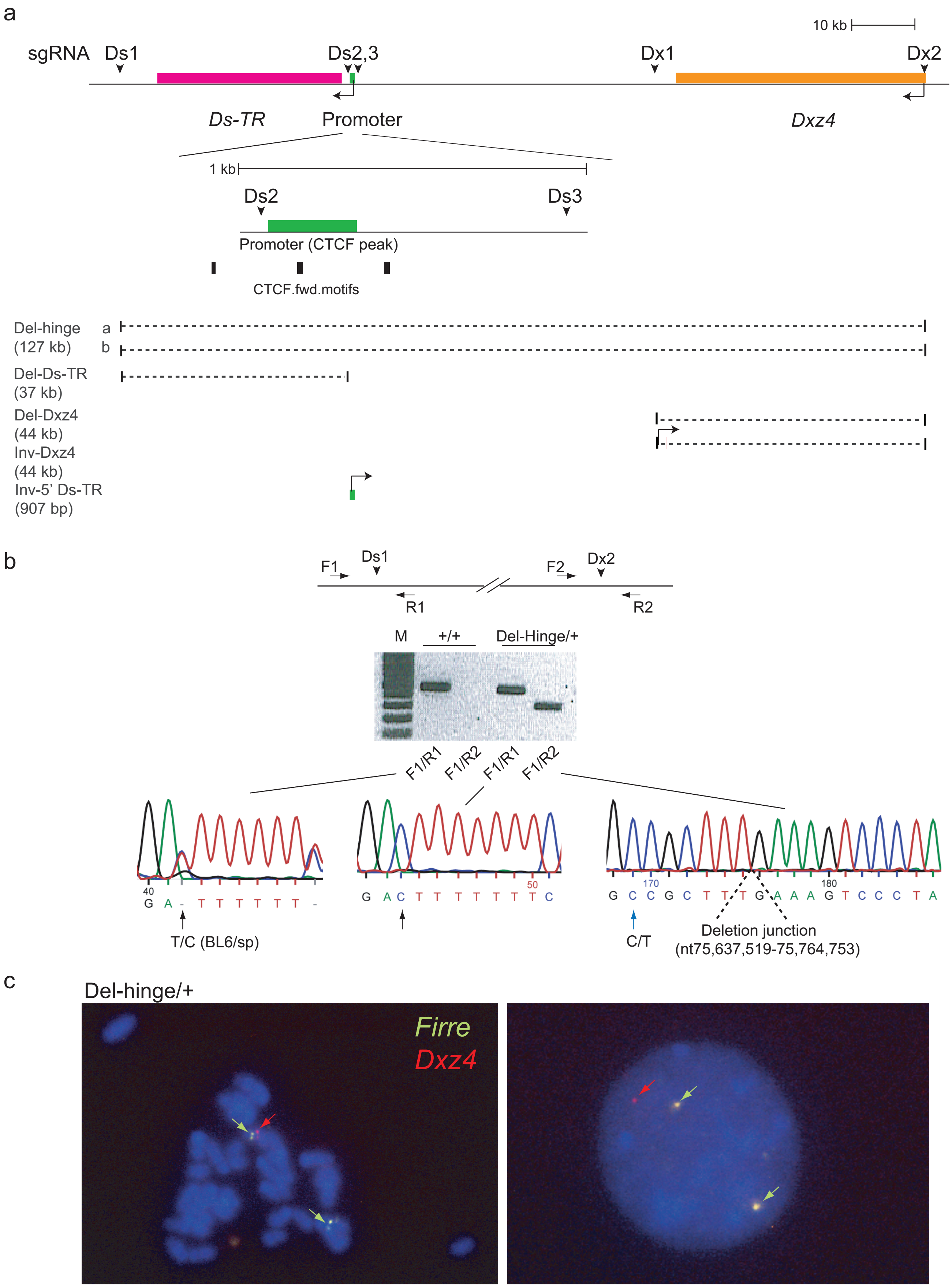
a
10 kb
sgRNA
Ds1
Ds2,3
Dx1
Dx2
Ds-TR
Promoter
Dxz4
1 kb
Ds2
Ds3
Promoter (CTCF peak)
CTCF.fwd.motifs
Del-hinge (127 kb)
a
b
Del-Ds-TR (37 kb)
Del-Dxz4 (44 kb)
Inv-Dxz4 (44 kb)
Inv-5' Ds-TR (907 bp)
b
F1
Ds1
F2
Dx2
R1
R2
M
+/+
Del-Hinge/+
F1/R1
F1/R2
F1/R1
F1/R2
40
G A - T T T T T T -
T/C (BL6/sp)
50
G A C T T T T T T T C
170
180
G C C G C T T T G A A A G T C C C T A
C/T
Deletion junction
(nt75,637,519-75,764,753)
c
Del-hinge/+
Firre
Dxz4

Supplementary Figure 1, Related to Figure 1.
Generation and verification of the deletions and inversions of the mouse Xi. **a**. Schematic of the hinge region indicating the position of the guide RNAs used for CRISPR/Cas9 editing. Deletion of the whole hinge (127kb, nt75637519-75764753) was obtained in two independent experiments using Ds1 and Dx2 and includes the transcriptional start site of the *Dxz4*-associated lncRNA gene *4933407K13Rik*; Deletion of *Ds-TR* (37kb, nt75637501-75674037) was obtained using Ds1 and Ds2 and does not include the promoter region of *Ds-TR*; Inversion (907bp, nt75674046-75674952) of 2 of 3 CTCF binding sites located 5' of *Ds-TR* was obtained using Ds2 and Ds3; Deletion and inversion of *Dxz4* (44kb, nt75721096-75764754) were obtained using Dx1 and Dx2 (Supplementary Table 6). **b**. Example of a verification of one of the alterations: PCR amplification using primers F1 and R1 followed by Sanger sequencing verified loss of SNPs from the BL6 allele and PCR amplification using primers F1 and R2 revealed the new junction sequence (Supplementary Table 7). **c**. Deletion of the hinge was also verified by fluorescence in situ hybridization (FISH) using a BAC probe for *Dxz4* (red) and a control BAC probe for *Firre* (green). Left, example of a metaphase chromosome preparation with one intact X chromosome with green and red signals (arrows) and a *Dxz4*-deleted X chromosome with only a green signal (arrow); Right, example of a nucleus with one red (*Dxz4*) and two green control signals (arrows).

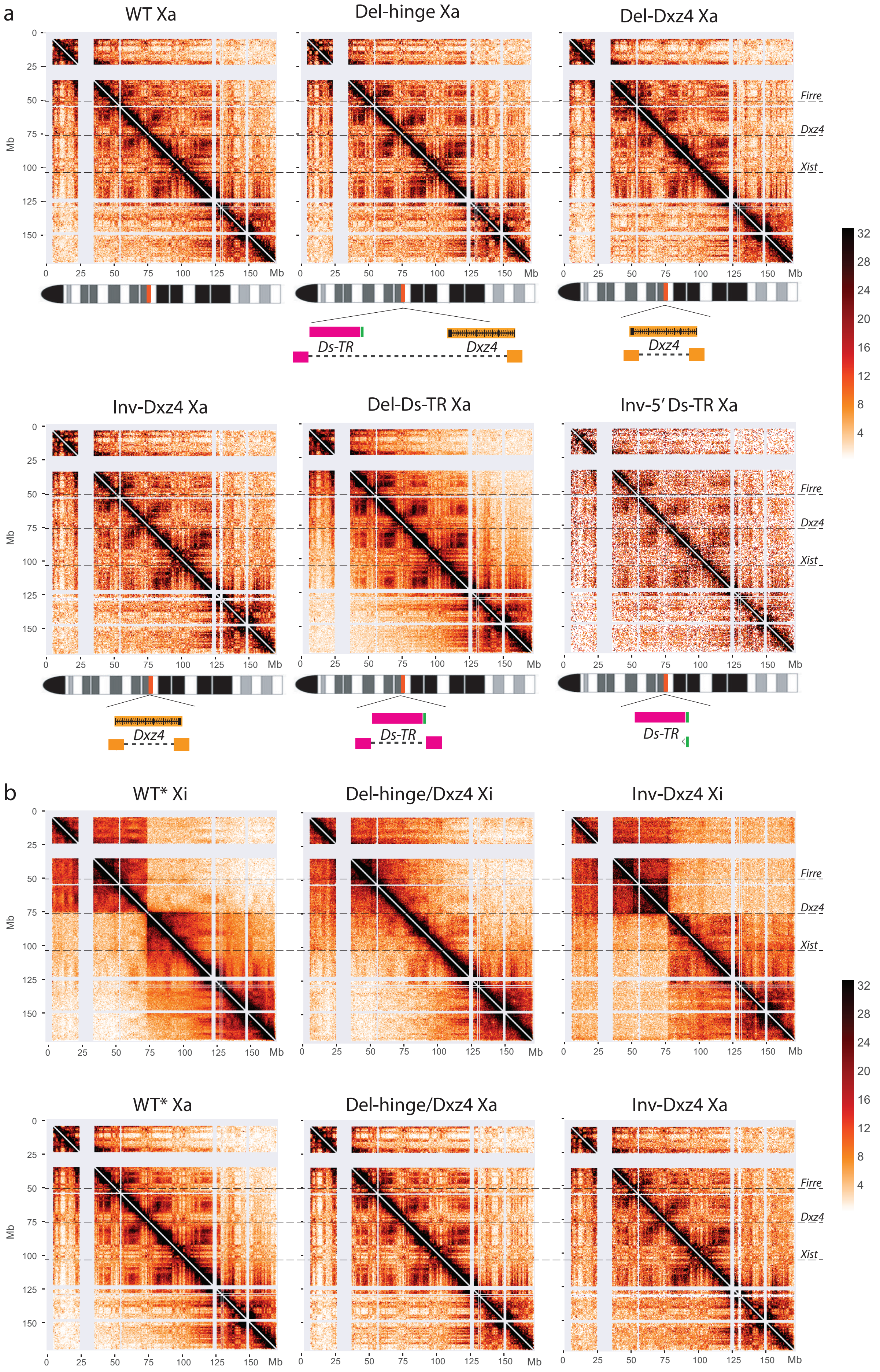

a
WT Xa
Del-hinge Xa
Del-Dxz4 Xa
Inv-Dxz4 Xa
Del-Ds-TR Xa
Inv-5′ Ds-TR Xa
Mb
Firre
Dxz4
Xist
Ds-TR
Dxz4
b
WT* Xi
Del-hinge/Dxz4 Xi
Inv-Dxz4 Xi
WT* Xa
Del-hinge/Dxz4 Xa
Inv-Dxz4 Xa
32
28
24
20
16
12
8
4

Supplementary Figure 2, Related to Figure 1.

Allelic contact maps for the X chromosome. **a**. Contact maps for the Xa do not differ between WT and Del-hinge, Del-Dxz4, Inv-Dxz4, Del-Ds-TR, and Inv-5'Ds-TR. Contact maps are shown at 500kb resolution. The position of *Dxz4* on the X and schematics of the allele-specific deletions/inversion are shown under the maps. The color scale reflects the normalized contact counts. **b.** Contact maps for the Xi and Xa representing two pooled sets of Hi-C contacts: one data set representing wild-type and Del-Ds-TR (WT*), and the other representing Del-hinge and Del-Dxz4 (Del-hinge/Dxz4). Allelic contact maps for each pool are very similar to those obtained from the pooled data. Allelic contacts maps for the Inv-Dxz4 Xi and Xa (duplicated from Fig. 1 and panel a above, respectively) are included to facilitate direct comparisons. Contact maps were generated at 500kb resolution. The location of *Dxz4*, *Firre* and *Xist* and schematics of the allele-specific deletions/inversions are shown. The color scale reflects normalized contact counts.

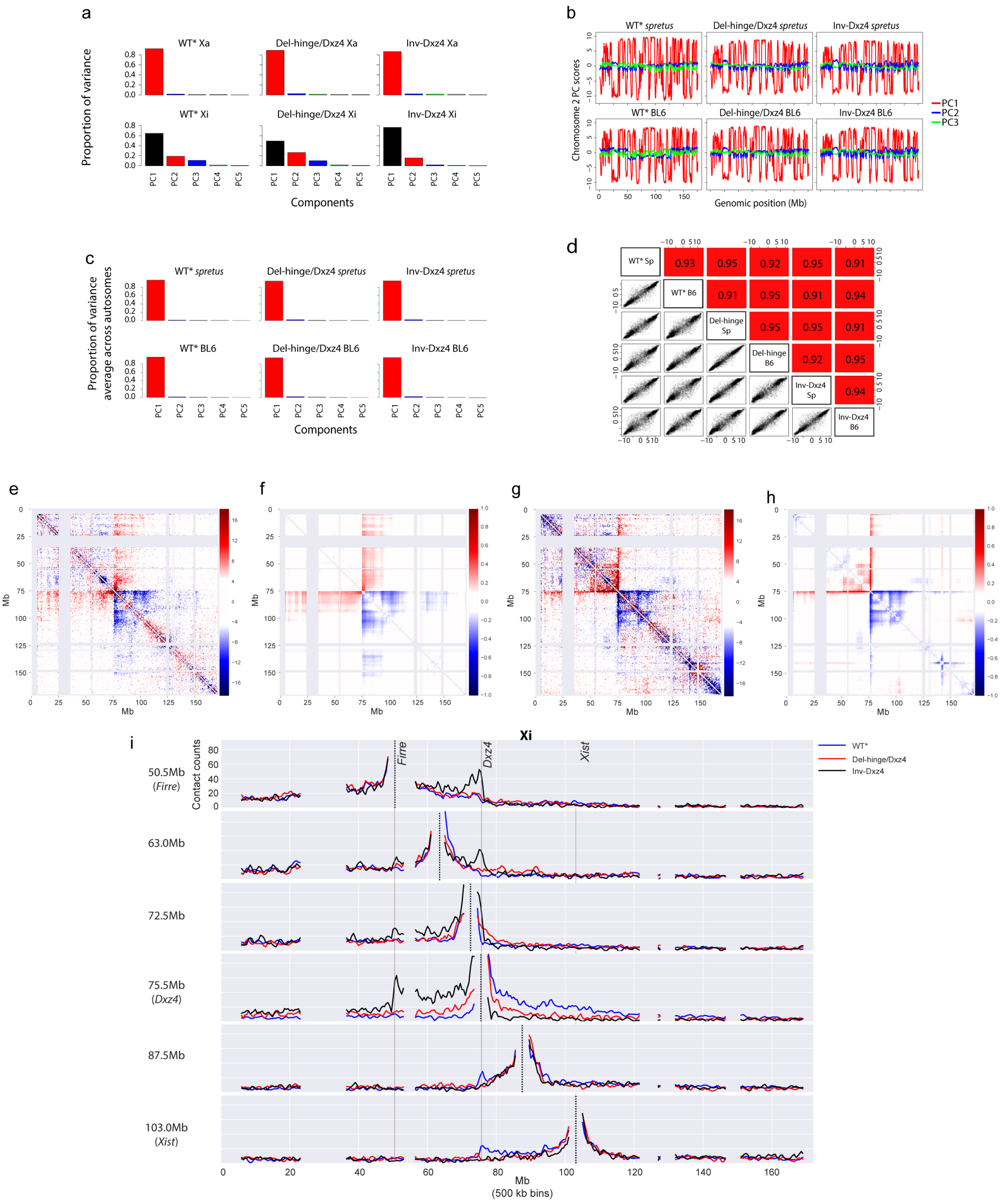
a
Proportion of variance
WT* Xa
Del-hinge/Dxz4 Xa
Inv-Dxz4 Xa
WT* Xi
Del-hinge/Dxz4 Xi
Inv-Dxz4 Xi
PC1 PC2 PC3 PC4 PC5
Components
b
WT* spretus
Del-hinge/Dxz4 spretus
Inv-Dxz4 spretus
WT* BL6
Del-hinge/Dxz4 BL6
Inv-Dxz4 BL6
Chromosome 2 PC scores
Genomic position (Mb)
PC1
PC2
PC3
c
Proportion of variance average across autosomes
WT* spretus
Del-hinge/Dxz4 spretus
Inv-Dxz4 spretus
WT* BL6
Del-hinge/Dxz4 BL6
Inv-Dxz4 BL6
Components
d
WT* Sp
WT* B6
Del-hinge Sp
Del-hinge B6
Inv-Dxz4 Sp
Inv-Dxz4 B6
0.93 0.95 0.92 0.95 0.91
0.91 0.95 0.91 0.94
0.95 0.95 0.91
0.92 0.95
0.94
e
f
g
h
Mb
i
Xi
Contact counts
50.5Mb (Firre)
63.0Mb
72.5Mb
75.5Mb (Dxz4)
87.5Mb
103.0Mb (Xist)
Firre
Dxz4
Xist
WT*
Del-hinge/Dxz4
Inv-Dxz4
Mb (500 kb bins)

Supplementary Figure 3, Related to Figures 2 and 3.
Allelic principal component (PC) analyses (a-d), differential contact maps (e-h), and 4C analyses (i). **a.** The variance in the PC score profiles for the X chromosomes is explained by the top five allelic principal components for the Xa and Xi for WT*, Del-hinge/Dxz4, and Inv-Dxz4. **b.** WT*, Del-hinge/Dxz4, and Inv-Dxz4 allelic PC score profiles for an exemplar autosome (chr. 2) based on distance-corrected, normalized contact maps with counts binned at 500kb resolution (*spretus* top row; BL6 bottom row). In each case, the top three allelic PC scores are shown (red, blue and green, respectively). **c.** The variance is explained by the top five allelic principal components averaged across all autosomes (*spretus* top row; BL6 bottom row) for WT*, Del-hinge/Dxz4, and Inv-Dxz4. **d.** Pairwise Spearman correlation values and associated scatterplots between allelic PC1 scores for autosomes concatenated end-to-end for WT*, Del-hinge/Dxz4, and Inv-Dxz4. **e**. Differential contact map based on untransformed count data at 500kb resolution to highlight differences between Del-hinge/Dxz4 Xi and WT* Xi (loss or gain of contacts in the Del-hinge/Dxz4 versus WT* appear blue or red, respectively). Color scale shows differential normalized contact counts. **f**. As in (e) for differential contact map based on Pearson correlation transformed data. Color scale shows differential Pearson correlation values. **g**. As in (e) to highlight differences between Inv-Dxz4 Xi and WT* Xi. **h**. As in (g) for differential contact map based on Pearson correlation transformed data. **i**. Virtual 4C plots derived from the Hi-C data for various 500kb viewpoints positioned along the Xi for WT* (blue), Del-hinge/Dxz4 (red), and Inv-Dxz4 (black). Y-axis (contact counts) limited to 20% of maximum. The position of the viewpoints is indicated.

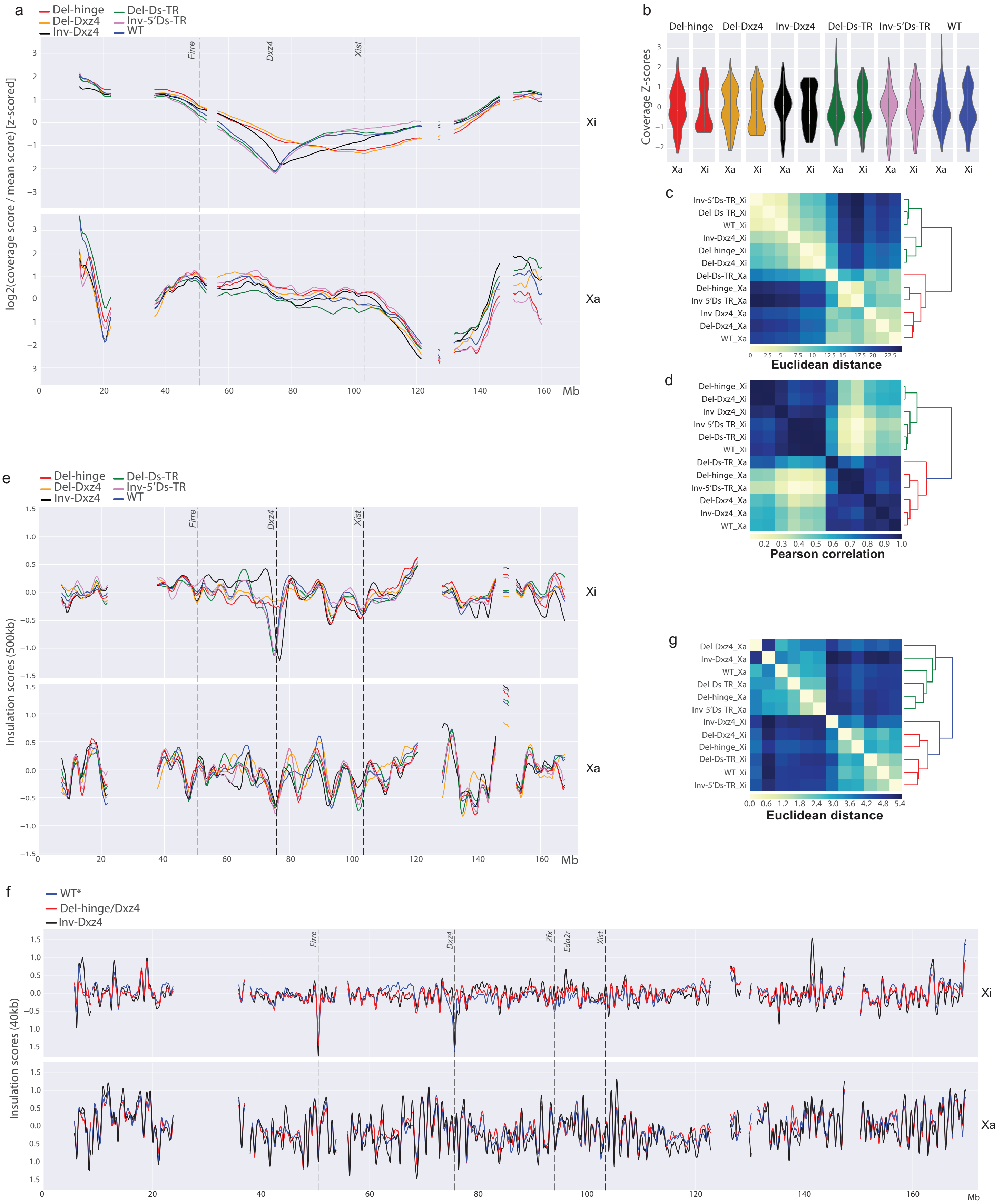
a
Del-hinge
Del-Dxz4
Inv-Dxz4
Del-Ds-TR
Inv-5′Ds-TR
WT
Firre
Dxz4
Xist
Xi
Xa
log2(coverage score / mean score) [z-scored]
Mb
b
Del-hinge Del-Dxz4 Inv-Dxz4 Del-Ds-TR Inv-5′Ds-TR WT
Coverage Z-scores
Xa Xi Xa Xi Xa Xi Xa Xi Xa Xi Xa Xi
c
Inv-5′Ds-TR_Xi
Del-Ds-TR_Xi
WT_Xi
Inv-Dxz4_Xi
Del-hinge_Xi
Del-Dxz4_Xi
Del-Ds-TR_Xa
Del-hinge_Xa
Inv-5′Ds-TR_Xa
Inv-Dxz4_Xa
Del-Dxz4_Xa
WT_Xa
Euclidean distance
d
Del-hinge_Xi
Del-Dxz4_Xi
Inv-Dxz4_Xi
Inv-5′Ds-TR_Xi
Del-Ds-TR_Xi
WT_Xi
Del-Ds-TR_Xa
Del-hinge_Xa
Inv-5′Ds-TR_Xa
Del-Dxz4_Xa
Inv-Dxz4_Xa
WT_Xa
Pearson correlation
e
Del-hinge
Del-Dxz4
Inv-Dxz4
Del-Ds-TR
Inv-5′Ds-TR
WT
Firre
Dxz4
Xist
Xi
Xa
Insulation scores (500kb)
Mb
g
Del-Dxz4_Xa
Inv-Dxz4_Xa
WT_Xa
Del-Ds-TR_Xa
Del-hinge_Xa
Inv-5′Ds-TR_Xa
Inv-Dxz4_Xi
Del-Dxz4_Xi
Del-hinge_Xi
Del-Ds-TR_Xi
WT_Xi
Inv-5′Ds-TR_Xi
Euclidean distance
f
WT*
Del-hinge/Dxz4
Inv-Dxz4
Firre
Dxz4
Zfx
Eda2r
Xist
Xi
Xa
Insulation scores (40kb)
Mb

Supplementary Figure 4, Related to Figures 3 and 4.
Coverage (a-d) and insulation score analyses (e-g). **a**. Standardized coverage Z-score profiles using 500kb resolution Hi-C contact map data along the Xi (top) and Xa (bottom) in WT (blue), Del-hinge (red), Del-Dxz4 (orange), Inv-Dxz4 (black), Del-Ds-TR (green), and Inv-5'Ds-TR cells (indigo). The positions of *Firre, Dxz4,* and *Xist* are indicated. **b**. Violin plots showing the distribution of standardized coverage Z-scores using 500kb bins for the Xi and Xa in WT (blue), Del-hinge (red), Del-Dxz4 (orange), Inv-Dxz4 (black), Del-Ds-TR (green), and Inv-5'Ds-TR cells (indigo). Box and whisker demarcations as described for Fig. 4g. **c**. Hierarchical clustering based on the Euclidean distance between standardized coverage Z-scores using 500kb bins along the Xi and Xa in WT, Del-hinge, Del-Dxz4, Inv-Dxz4, Del-Ds-TR, and Inv-5'Ds-TR cells. **d**. Hierarchical clustering based on Pearson correlation (using 1 - *r* as the distance measure) for standardized coverage Z-scores using 500kb bins along the Xi and Xa in WT, Del-hinge, Del-Dxz4, Inv-Dxz4, Del-Ds-TR, and Inv-5'Ds-TR cells. **e**. As in (a) for insulation scores. **f**. As in (c) for insulation scores. **g**. Profiles of insulation scores using 40kb bins along the entire length of the Xi (top) and Xa (bottom) in WT* (blue), Del-hinge/Dxz4 (red), and Inv-Dxz4 (black). The positions of *Firre, Dxz4, Zfx, Eda2r,* and *Xist* are indicated.

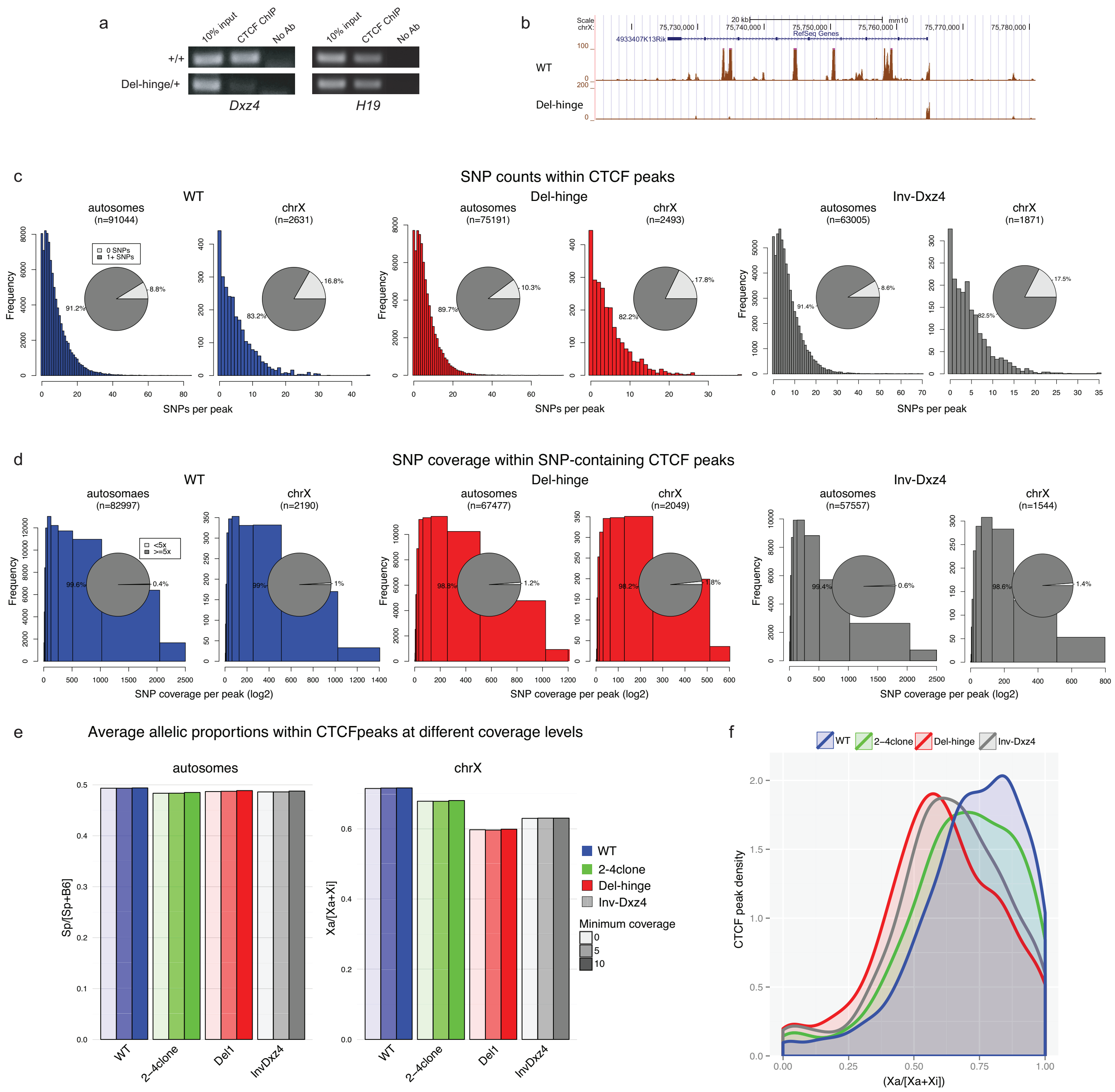

Supplementary Figure 5, Related to Figure 6.

CTCF ChIP-seq SNP coverage and allelic proportion. **a**. PCR amplification of *Dxz4* and a control imprinted region near the autosomal *H19* gene using the input fraction (10% input), the ChIP fraction (CTCF ChIP) and the no antibody fraction (No Ab) for WT cells (+/+) and Del-hinge cells (Del-hinge/+). A strong decrease in CTCF enrichment at *Dxz4* is seen in Del-hinge due to deletion of *Dxz4* on the Xi, which normally binds CTCF. **b**. CTCF binding to *Dxz4* on the Xi is lost in Del-hinge, as shown on genome browser view of ChIP-seq. **c**. Histograms of SNP counts within CTCF peaks along autosomes and the X-chromosomes in WT (blue), Del-hinge (red), and Inv-Dxz4 (grey). **d**. Histograms of the SNP read coverage within SNP-containing CTCF peaks along autosomes and the X chromosomes in WT (blue), Del-hinge (red) and Inv-Dxz4 (grey). **e**. Overall allelic proportion across all CTCF peaks at three different levels of read coverage (0, 5x, 10x) along autosomes and the X-chromosomes in WT (blue), Patski2-4 (green), Del-hinge (red) and Inv-Dxz4 (grey). See additional analyses in Supplementary Fig. 6. **f**. Density histograms of the distribution of allelic proportions of CTCF peaks (Xa/(Xa+Xi) along the X-chromosomes for WT (blue), Patski2-4 (green), Del-hinge (red) and Inv-Dxz4 (grey). A shift in the distribution of allelic proportions due to an increase in CTCF binding on the Xi is evident for the X chromosome in Del-hinge and to a lesser extent Inv-Dxz4, compared to WT and Patski2-4.

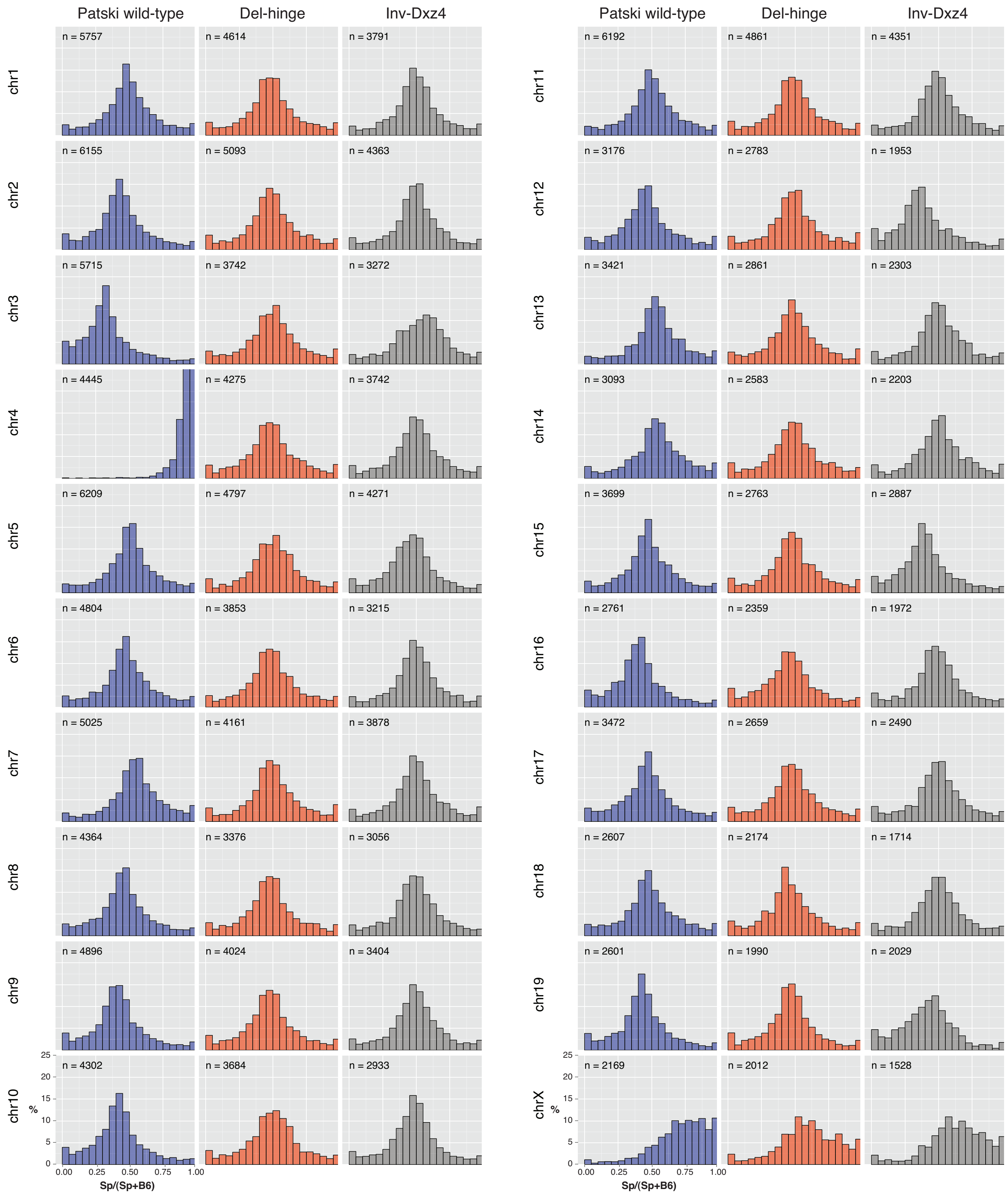

Supplementary Figure 6, Related to Figure 6.

Histograms showing the percentage of peaks exhibiting the specified allelic proportions of CTCF peaks (*spretus*/(*spretus* +BL6)) per chromosome for WT (blue), Del-hinge (red) and Inv-Dxz4 (grey). Y-axes have a maximum value of 25%. Skewing in the distributions of chromosomes 3 and 4 in WT due to known BL6 trisomy 3 and monosomy 4 in a high proportion of WT cells, not present in Del-hinge and Inv-Dxz4 clones.

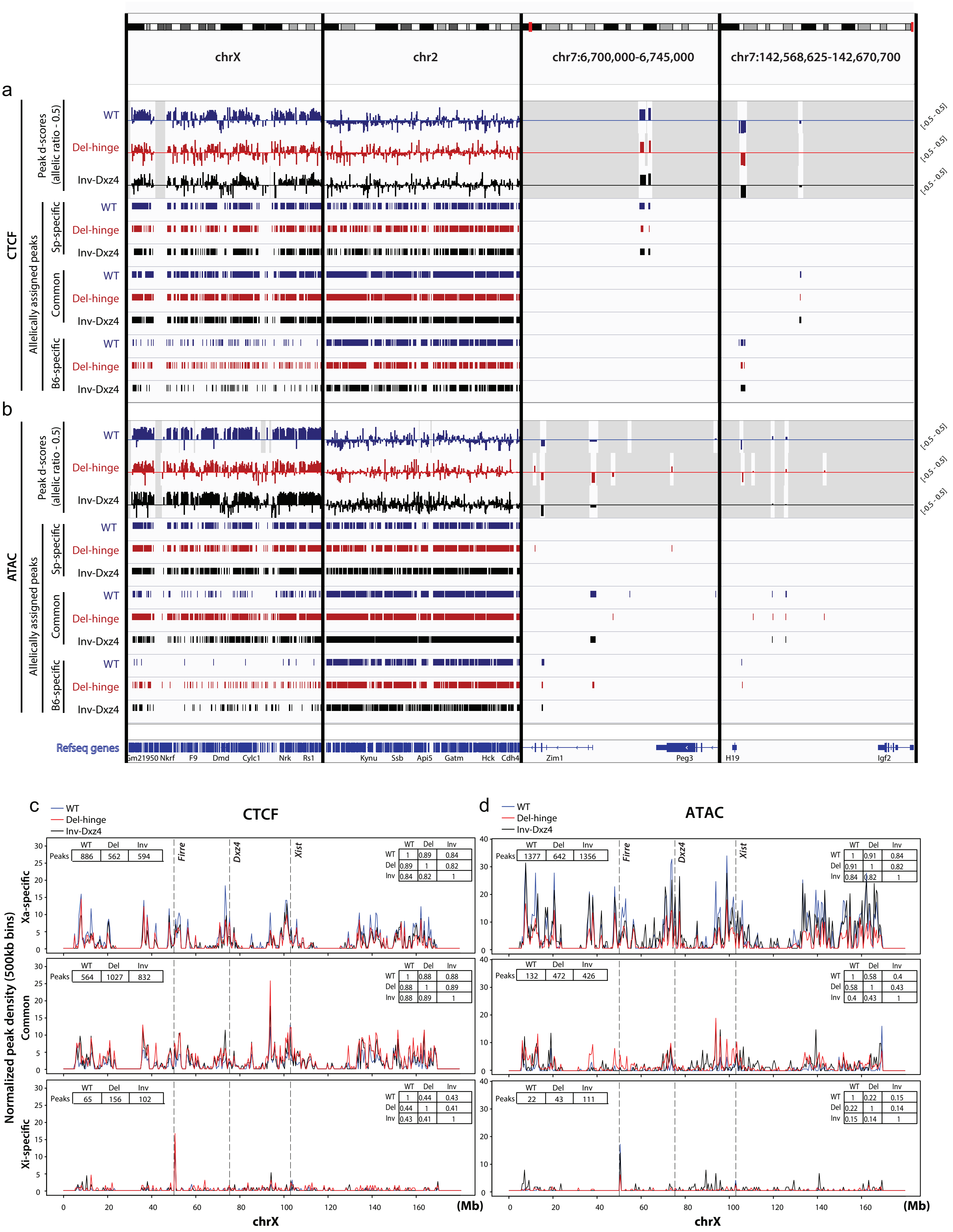
a
b
c
d
chrX
chr2
chr7:6,700,000-6,745,000
chr7:142,568,625-142,670,700
CTCF
ATAC
Peak d-scores (allelic ratio - 0.5)
Allelically assigned peaks
Sp-specific
Common
B6-specific
WT
Del-hinge
Inv-Dxz4
[-0.5 - 0.5]
Refseq genes
Gm21950 Nkrf F9 Dmd Cylc1 Nrk Rs1
Kynu Ssb Api5 Gatm Hck Cdh4
Zim1
Peg3
H19
Igf2
CTCF
ATAC
Normalized peak density (500kb bins)
Xa-specific
Common
Xi-specific
Firre
Dxz4
Xist
Peaks
WT Del Inv
886 562 594
564 1027 832
65 156 102
1377 642 1356
132 472 426
22 43 111
chrX
(Mb)

Supplementary Figure 7, Related to Figures 6, 7, 9.
CTCF and ATAC browser tracks (a, b) and peak location (c, d). **a**. Genome browser tracks of CTCF ChIP-seq peak d-scores ((*spretus*/(*spretus* +BL6) - 0.5) and of peaks assigned as *spretus*-specific, common, or BL6-specific peaks along the entire X chromosome, and exemplar autosome (chromosome 2), as well as around two imprinted genes (*Peg3*, *H19*) on chromosome 7 in WT (blue), Del-hinge (red) and Inv-Dxz4 (black). **b**. As in (a) but for ATAC-seq data. **c.** Plots of Xa-specific, common, and Xi-specific CTCF peak density (counts binned within 500kb windows) along the X chromosome for WT (blue), Del-hinge (red), and Inv-Dxz4 (black). To account for differences in the number of SNP-covered peaks obtained between samples (WT, Del-hinge, and Inv-Dxz4) due to differences in the depth of sequencing (Supplementary Tables 3 and 4), the binned counts are scaled by a factor obtained from the between-sample ratios of autosomal diploid SNP-covered peaks. The scaling factors were 0.7 and 0.87 and 1 for WT, Del-hinge, and Inv-Dxz4, respectively. The tables in the top left corner show the total number of peaks under the curves after normalization and those in the top right corner are Spearman correlation matrices across samples (Del: Del-hinge; Inv: Inv-Dxz4). The positions of *Firre, Dxz4,* and *Xist* are indicated. **d.** As in (c) but for ATAC peaks, but the scaling factors were 0.5 and 0.38 and 1 for WT, Del-hinge, and Inv-Dxz4, respectively.

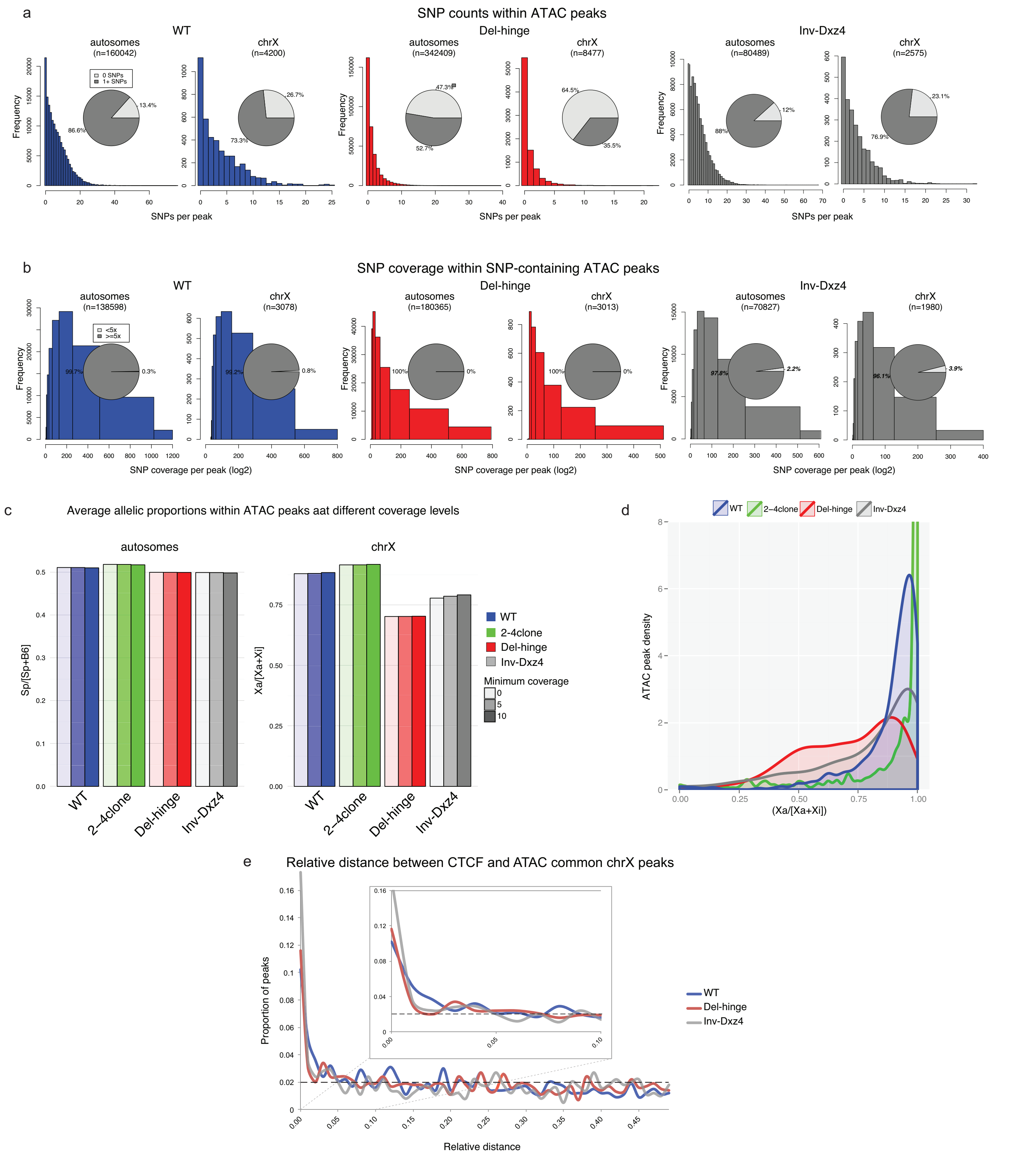

Supplementary Figure 8, Related to Figure 7.
ATAC-seq SNP coverage and allelic proportion. **a-d.** As is Supplementary Fig. 5c-d but for ATAC peaks, except that in panel d the maximum Y axis value was set to 8. See additional analyses in Supplementary Fig. 9. **e.** Plot of the proportions of common CTCF peaks exhibiting the range of relative distances ([0; 0.5]) to common ATAC peaks along the x-axis for WT (blue), Del-hinge (red), and Inv-Dxz4 (grey). The dashed horizontal line at 0.02 represents the uniformly distributed proportion if there were no spatial correlation between the two sets of peaks. Inset: As in (e), but for the relative distance range [0; 0.1] to better show that observed relative distances are shifted towards low values.

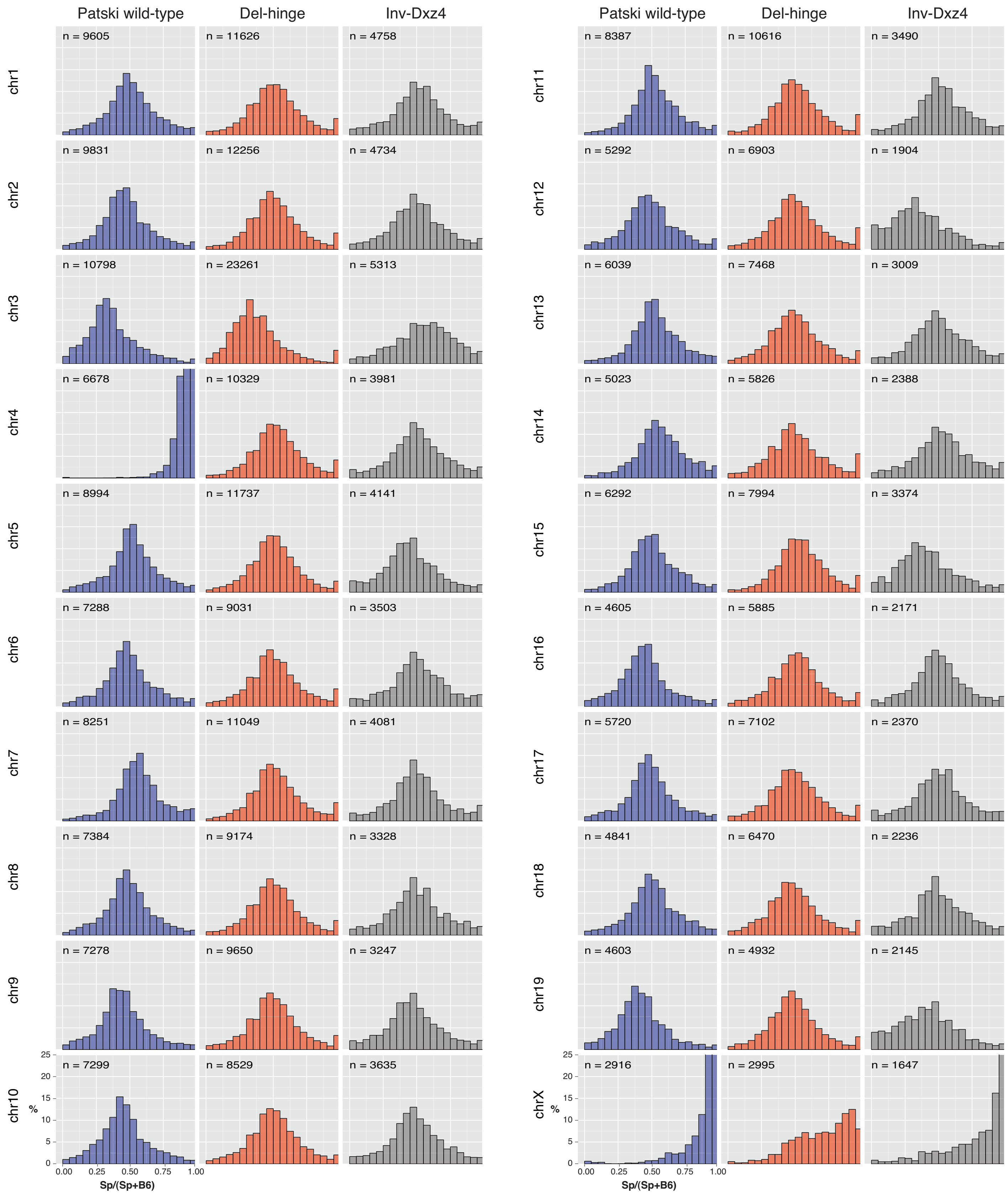

Supplementary Figure 9, Related to Figure 7.
As in Supplementary Figure 6 but for ATAC peaks.

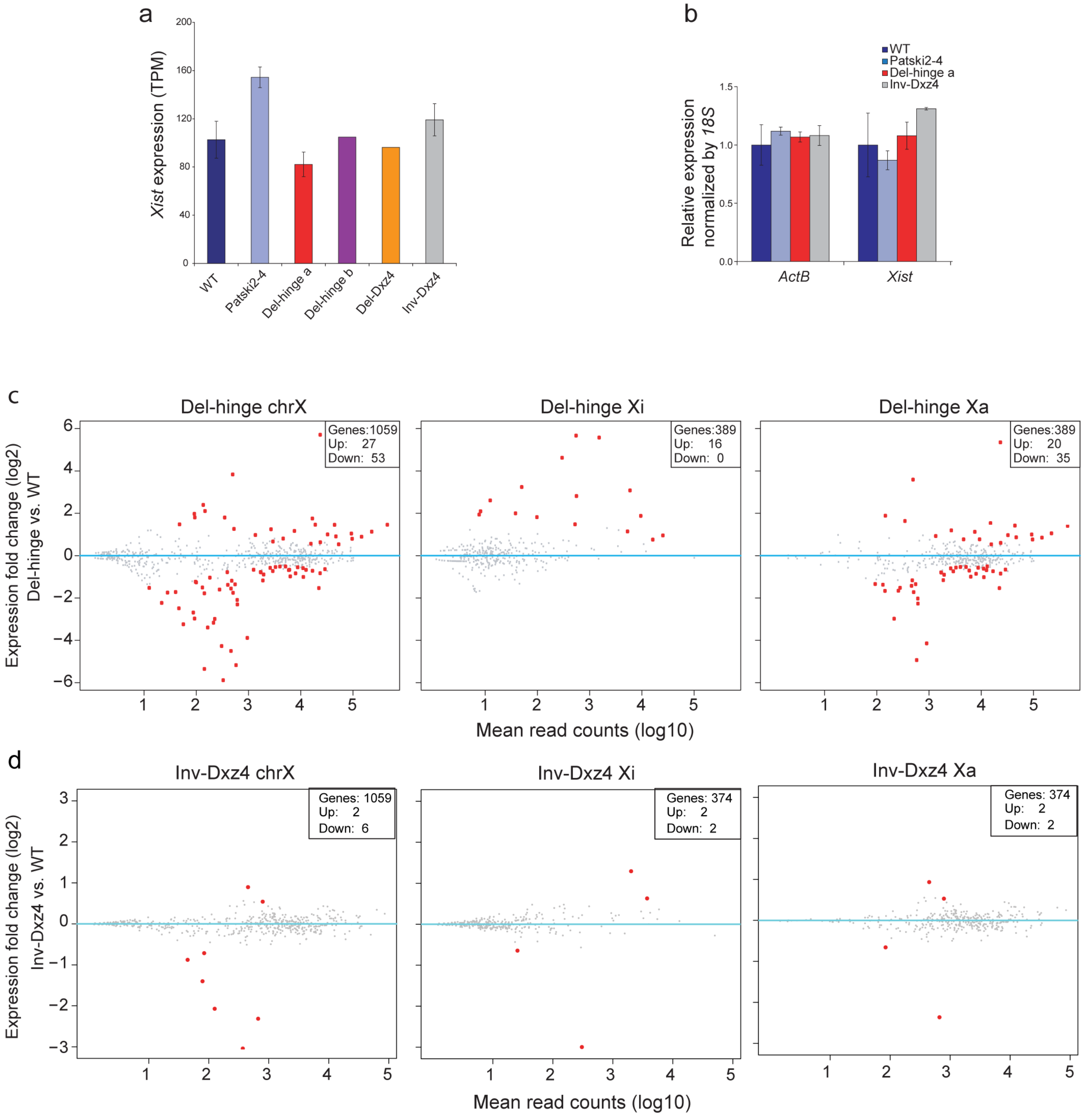

Supplementary Figure 10, Related to Figures 8 and 9.
No change of *Xist* expression (a, b) and few changes in X-linked gene expression overall upon deletion or inversion of *Dxz4* (c, d). **a.** Expression levels of *Xist* from RNA-seq in WT, Patski2-4, Del-hinge clone a and b, and Inv-Dxz4 are similar. **b.** Quantitative RT-PCR confirms similar *Xist* expression levels in WT, Patski2-4, Del-hinge clone a and Inv-Dxz4. No significant change was detected (*p*-value >0.05 from two-tail Student's *t*-test). Error bars, s.e.m. *18S* was used for normalization and *ActB* as a control gene. **c.** Plots of expression fold-changes (log2) between Del-hinge versus WT for transcripts on the X chromosome and for subsets of transcripts specifically from the Xi and Xa, based on SNPs, relative to expression levels (mean read counts). The total number of genes examined and the number of genes that show significantly increased expression (Up) or lower expression (Down) in Del-hinge versus WT is indicated in the box (log2 fold change > 0.5 and adjusted *p*-value < 0.05 by the Wald test used by DESeq2). Grey dots represent genes and red dots indicate genes with significantly changed expression. **d**. As in (c) but for expression fold-changes (log2) between Inv-Dxz4 versus wild-type Patski2-4. See additional analyses in Supplementary Table 7, Supplementary Data 2-5 and Supplementary Fig. 11.

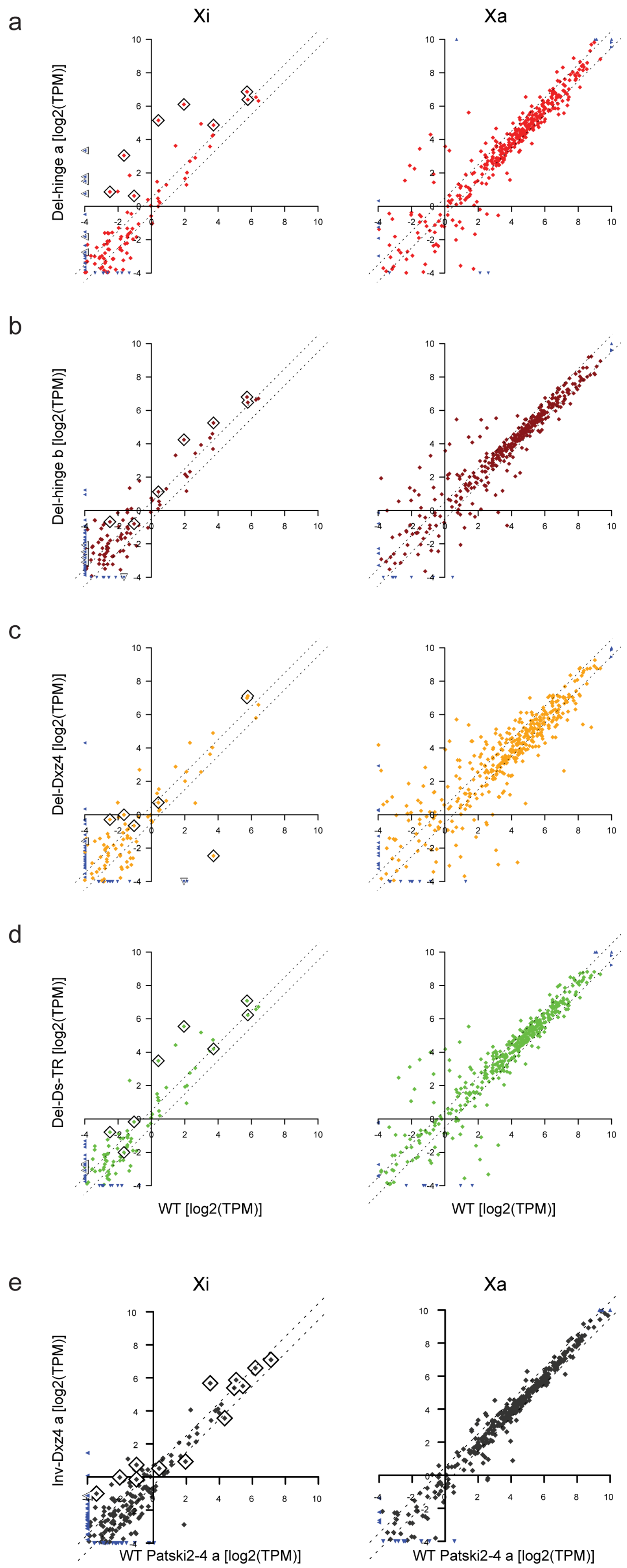
a
Xi
Xa
Del-hinge a [log2(TPM)]
b
Del-hinge b [log2(TPM)]
c
Del-Dxz4 [log2(TPM)]
d
Del-Ds-TR [log2(TPM)]
WT [log2(TPM)]
WT [log2(TPM)]
e
Xi
Xa
Inv-Dxz4 a [log2(TPM)]
WT Patski2-4 a [log2(TPM)]
WT Patski2-4 a [log2(TPM)]

Supplementary Figure 11, Related to Figure 9.

Gene expression changes in Del-hinge clone a **(a)**, Del-hinge clone b **(b)**, Del-Dxz4 **(c)**, and Del-Ds-TR **(d)** compared to WT cells, and in Inv-Dxz4 versus wild-type Patski2-4 (**e**). Scatter plots of Xi- and Xa-specific expression between each deleted clone and WT are shown for genes with mean log2(TPM) ≥ -4 diploid (i.e. mean TPM ≥ 0.0625). Dot lines represent 1.5-fold cutoffs. Genes marked by a diamond represent the 16 genes that showed a significant change in Xi-specific expression between WT and Del-hinge clone a. Note that only genes expressed at least in one condition are plotted. See additional analyses in Supplementary Table 7 and Supplementary Data 2-5.

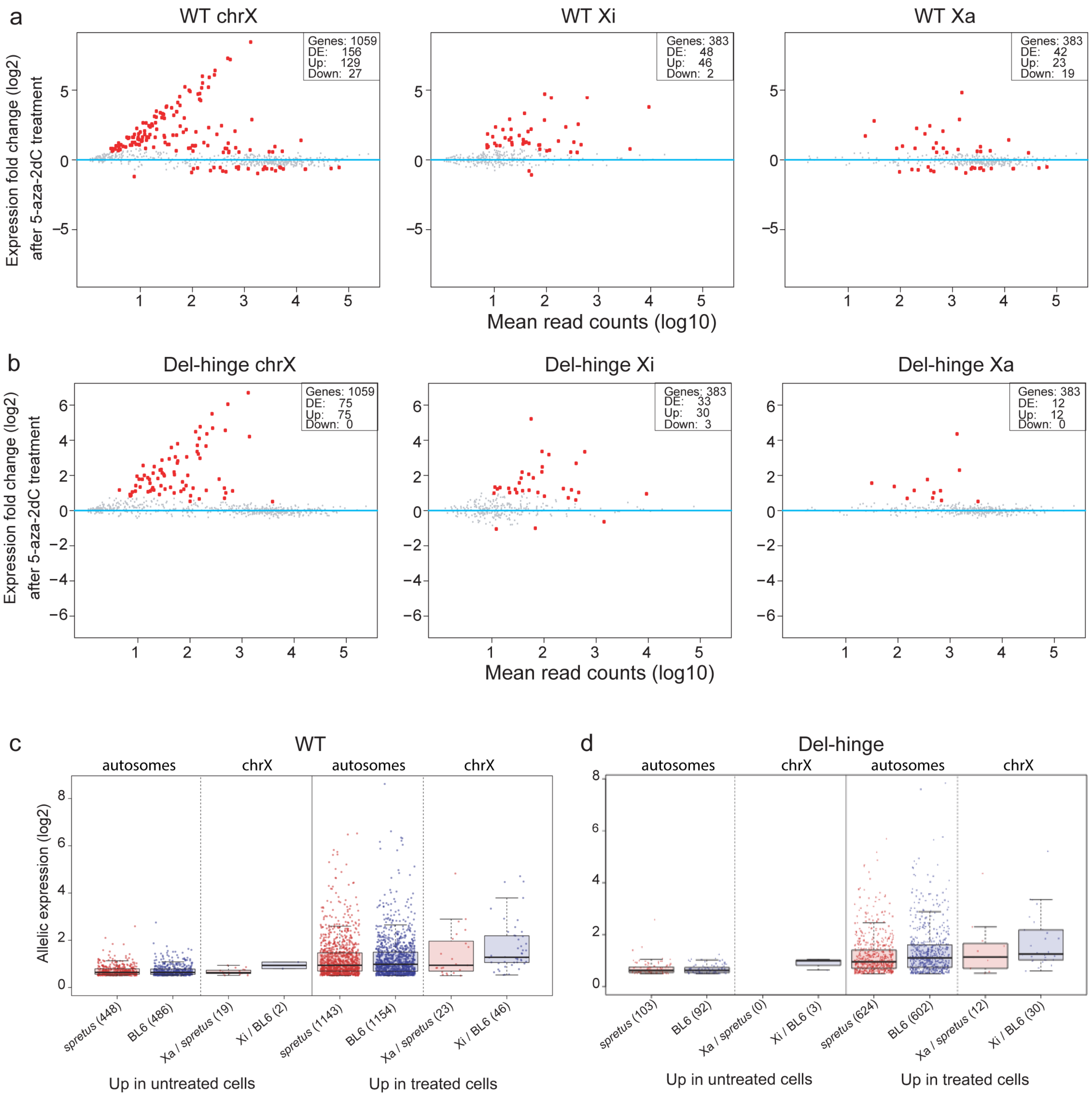

Supplementary Figure 12, Related to Figure 9.

Gene expression changes in WT and Del-hinge clone a after 5-aza-2dC treatment. **a**, **b**. Plots of expression fold-changes (log2) between 5-aza-2dC -treated cells and controls in WT (a) and Del-hinge clone a (b) for all transcripts on the X chromosome (chrX) and for allele-specific transcripts from the Xi and Xa, relative to expression levels (mean read counts). The total number of genes examined and the number of genes that show significantly increased expression (Up), decreased expression (Down) and the total number of genes with differential expression (DE) in treated versus untreated cells are indicated in the box. (log2 fold change >0.5, adjusted *p*-value <0.05 by the Wald test used by DESeq2). Grey dots represent genes and red dots indicate genes with significantly changed expression. **c**, **d**. Box plots showing the distribution of allelic expression of genes with increased expression (Up) in untreated and 5-aza-2dC treated cells in WT (c) and Del-hinge clone a (d) for autosomal (A_*spretus* or A_BL6) and X-linked (Xa or Xi) genes. Red, *spretus* alleles; blue, BL6 alleles. See additional analyses in Supplementary Data 6 and 7. Box and whisker demarcations as described for Fig. 8c-f.

Supplementary Table 1: In situ DNase Hi-C valid read pairs before and after merging

<table>
<tr><th colspan="3">Unpooled (sequencing runs)</th><th colspan="4">Individual cell types</th><th colspan="4">Pooled (WT* and Del-hinge/Dxz4/Dxz4)</th></tr>
<tr><th>Sequencing run</th><th>Read length (per end)</th><th>Valid read pairs</th><th>Sample</th><th>Valid read pairs</th><th>BL6-specific read pairs</th><th>spretus-specific read pairs</th><th>Pooled sample</th><th>Valid read pairs</th><th>BL6-specific read pairs</th><th>spretus-specific read pairs</th></tr>
<tr><td>Patski WT</td><td>150</td><td>31,017,733</td><td rowspan="13">WT</td><td rowspan="13">250,110,923</td><td rowspan="13">61,160,112</td><td rowspan="13">64,392,323</td><td rowspan="15">WT*</td><td rowspan="15">365,294,454</td><td rowspan="15">97,529,009</td><td rowspan="15">100,639,206</td></tr>
<tr><td>Patski WT</td><td>150</td><td>30,791,787</td></tr>
<tr><td>Patski WT</td><td>150</td><td>31,378,804</td></tr>
<tr><td>Patski WT</td><td>150</td><td>31,262,773</td></tr>
<tr><td>Patski WT</td><td>80</td><td>20,073,848</td></tr>
<tr><td>Patski WT</td><td>80</td><td>19,786,937</td></tr>
<tr><td>Patski WT</td><td>80</td><td>20,275,097</td></tr>
<tr><td>Patski WT</td><td>80</td><td>20,220,057</td></tr>
<tr><td>Patski WT</td><td>80</td><td>1,196,756</td></tr>
<tr><td>Patski WT</td><td>80</td><td>1,171,759</td></tr>
<tr><td>Patski WT</td><td>80</td><td>1,266,296</td></tr>
<tr><td>Patski WT</td><td>80</td><td>1,234,244</td></tr>
<tr><td>Patski WT</td><td>154</td><td>40,434,832</td></tr>
<tr><td>Patski Del-Ds-TR</td><td>80</td><td>101,918,089</td><td>Del-Ds-TR</td><td>101,918,089</td><td>31,534,068</td><td>31,292,655</td></tr>
<tr><td>Patski Inv-5' Ds-TR</td><td>154</td><td>13,265,442</td><td>Inv-5' Ds-TR</td><td>13,265,442</td><td>3,202,735</td><td>3,211,491</td></tr>
<tr><td>Patski Del-hinge</td><td>154</td><td>51,281,552</td><td rowspan="4">Del-hinge</td><td rowspan="4">184,240,459</td><td rowspan="4">30,200,476</td><td rowspan="4">30,762,468</td><td rowspan="5">Del-hinge/Dxz4</td><td rowspan="5">373,892,589</td><td rowspan="5">72,092,628</td><td rowspan="5">74,850,687</td></tr>
<tr><td>Patski Del-hinge</td><td>154</td><td>48,652,938</td></tr>
<tr><td>Patski Del-hinge</td><td>150</td><td>63,931,278</td></tr>
<tr><td>Patski Del-hinge</td><td>150</td><td>20,374,691</td></tr>
<tr><td>Patski Del-Dxz4</td><td>150</td><td>189,652,130</td><td>Del-Dxz4</td><td>189,652,130</td><td>40,184,143</td><td>42,218,639</td></tr>
<tr><td>Patski Inv-Dxz4</td><td>150</td><td>158,673,578</td><td>Inv-Dxz4</td><td>158,673,578</td><td>27,059,801</td><td>27,641,615</td><td></td><td></td><td></td><td></td></tr>
</table>

The number of valid read pairs and their length is listed for each Hi-C library together with the calculated total number of reads for each individual cell type, and for the pooled data sets (WT* and Del-hinge/Dxz4). Reads were mapped to mm10 using BWA MEM. The number of BL6-specific and *spretus*-specific interactions based on one unambiguously mapped end and discarding interactions < 20kb are listed.

Supplementary Table 2: Summary of TAD counts

bin size (kb)	cell line	X-allele	TADs
500	Patski WT*	Xi	14
	Patski Del-hinge/Dxz4	Xi	16
	Patski Inv-Dxz4	Xi	17
	Patski WT*	Xa	17
	Patski Del-hinge/Dxz4	Xa	21
	Patski Inv-Dxz4	Xa	17
40	Patski WT*	Xi	117
	Patski Del-hinge/Dxz4	Xi	139
	Patski Inv-Dxz4	Xi	125
	Patski WT*	Xa	131
	Patski Del-hinge/Dxz4	Xa	127
	Patski Inv-Dxz4	Xa	135

The estimated number of TADs is shown for the Xi and Xa for each cell line using different bin size.

Supplementary Table 3: Summary of CTCF ChIP-seq read counts and peaks

			WT		Del-hinge		Inv-Dxz4		Patski2-4	
			Counts	%	Counts	%	Counts	%	Counts	%
	Read pairs	Sequenced IP reads (2 x 75bp)	115,062,412		74,218,646		81,267,787		126,182,956	
		Sequenced input reads (2 x 75bp)	159,112,872		165,137,101		*Used Del-hinge input*		*Used Del-hinge input*	
		High-quality deduplicated primary mapped IP reads	39,335,415		22,741,658		24,260,327		36,793,673	
		High-quality deduplicated primary mapped input reads	65,615,369		56,188,199		*Used Del-hinge input*		*Used Del-hinge input*	
	Diploid peaks	genome-wide peaks	93,675		77,684		65,493		101,447	
		autosomal peaks	91,044		75,191		63,613		98,864	
		chrX peaks	2,631		2,493		1,880		2,583	
	SNP-containing peaks	genome-wide peaks	85,187	90.9%	69,526	89.5%	59,101	90.2%	91,419	90.1%
		autosomal peaks	82,997	91.2%	67,477	89.7%	57,557	90.5%	89,335	90.4%
		chrX peaks	2,190	83.2%	2,049	82.2%	1,544	82.1%	2,084	80.7%
Allelic peaks (% of SNP-containing peaks)	Genome	covered SNP peaks	84,863	99.6%	68,664	98.8%	59,355	99.3%	91,040	99.6%
		spretus-assigned	15,692	18.5%	10,205	14.9%	8,323	14.0%	14,233	15.6%
		BL6-assigned	14,482	17.1%	10,623	15.5%	9,056	15.3%	15,173	16.7%
		common	54,689	64.4%	47,836	69.7%	41,976	70.7%	61,634	67.7%
	Autosomes	covered SNP peaks	82,694	99.6%	66,652	98.8%	57,827	99.3%	88,969	99.6%
		spretus-assigned	14,422	17.4%	9,557	14.3%	7,729	13.4%	13,194	14.8%
		BL6-assigned	14,389	17.4%	10,443	15.7%	8,954	15.5%	15,062	16.9%
		common	53,883	65.2%	46,652	70.0%	41,144	71.2%	60,713	68.2%
	chrX	covered SNP peaks	2,169	99.0%	2,012	98.2%	1,528	98.5%	2,071	99.4%
		Xa-assigned	1,270	58.6%	648	32.2%	594	38.9%	1,039	50.2%
		Xi-assigned	93	4.3%	180	8.9%	102	6.7%	111	5.4%
		common	806	37.2%	1,184	58.8%	832	54.5%	921	44.5%

The number of read pairs is listed for the ChIP and the input in Patski WT and Del-hinge, as well as for the ChIP in Patski Inv-Dxz4 and the Patski2-4 clone. The number of diploid CTCF peaks and SNP-containing CTCF peaks including genome-wide, autosomal and X-linked peaks are listed together with the percentage in each category and the ratio between Del-hinge and WT. The number of allelic peaks including covered peaks, *spretus*- and BL6-assigned peaks and common peaks are listed together with the percentages in each category.

Supplementary Table 4: Summary of segregated CTCF ChIP-seq and input reads and allelic peak calls

		WT				Patski Del-hinge				Del-hinge/WT ratio*		
		spretus	BL6	Ambiguous	BL6/*spretus*	*spretus*	BL6	Ambiguous	BL6/*spretus*	*spretus*	BL6	Ambiguous
Deduplicated primary mapped reads	CTCF ChIP	9,124,976	8,165,500	21,054,943	0.89	4,985,098	4,568,454	12,352,327	0.92			
	Input	16,428,379	14,983,924	33,952,974	0.91	13,359,723	12,722,977	29,743,259	0.95			
Peaks	Genome	47,272	43,515	69,742	0.92	42,194	39,950	59,826	0.95	1.01	1.04	0.97
	chrX	1,472	709	2,247	0.48	1,372	949	2,272	0.69	1.05	1.51	1.14

Summary of segregated CTCF ChIP-seq and input reads and allelic peak numbers (genome-wide and along the X chromosome) called using those reads for WT and Del-hinge. The BL6/*spretus* ratio is given for each row and each sample, along with the Del-hinge/WT ratios of peak numbers. *The Del-hinge/WT peak abundance ratios were scaled by a correction factor based on the genome-wide WT/Del-hinge ratio of the total number of peaks (1.13).

Supplementary Table 5: Summary of ATAC-seq read counts and peaks

			WT		Del-hinge		Inv-Dxz4		Patski2-4	
			Counts	%	Counts	%	Counts	%	Counts	%
	Read pairs	sequence run1 (2 x 150bp)	32,714,426		63,548,820		129,505,499		278,027,006	
		sequence run2 (2 x 75 bp)	21,477,971		115,577,719		N/A		N/A	
		sequence run3 (2 x 75bp)	160,039,334		137,035,953		N/A		N/A	
		sequence run4 (2 x 75bp)	116,278,899		N/A		N/A		N/A	
		High-quality deduplicated primary mapped reads	51,886,726		130,045,772		21,983,451		45,046,809	
	Diploid peaks	genome-wide peaks	164,242		350,886		83,064		126,030	
		autosomal peaks	160,042		342,409		80,489		123,081	
		chrX peaks	4,200		8,477		2,575		2,949	
	SNP-containing peaks	genome-wide peaks	141,676	86.3%	183,378	52.3%	72,807	87.7%	102,030	81.0%
		autosomal peaks	138,598	86.6%	180,365	52.7%	70,827	88.0%	100,114	81.3%
		chrX peaks	3,078	73.3%	3,013	35.5%	1,980	76.9%	1,916	65.0%
Allelic peaks (% of SNP-containing peaks)	Genome	covered peaks	141,275	99.7%	183,355	100.0%	71,194	97.8%	101,690	99.7%
		spretus-assigned	27,546	19.5%	28,017	15.3%	13,447	18.9%	24,856	24.4%
		BL6-assigned	19,826	14.0%	26,351	14.4%	12,167	17.1%	20,202	19.9%
		common	93,903	66.5%	128,987	70.3%	45,580	64.0%	56,632	55.7%
	Autosomes	covered peaks	138,221	99.7%	180,343	100.0%	69,292	97.8%	99,784	99.7%
		spretus-assigned	24,800	17.9%	26,345	14.6%	12,082	17.4%	23,139	23.2%
		BL6-assigned	19,782	14.3%	26,239	14.5%	12,056	17.4%	20,146	20.2%
		common	93,639	67.7%	127,759	70.8%	45,154	65.2%	56,499	56.6%
	chrX	covered peaks	3,054	99.2%	3,012	100.0%	1,902	96.1%	1,906	99.5%
		Xa-assigned	2,746	89.9%	1,672	55.5%	1,365	71.8%	1,717	90.1%
		Xi-assigned	44	1.4%	112	3.7%	111	5.8%	56	2.9%
		common	264	8.6%	1,228	40.8%	426	22.4%	133	7.0%

As described in Supplementary Table 3, but for ATAC-seq.

Supplementary Table 6: Summary of RNA-seq read counts, alignment rates, and differential expression results

Cell type	sequenced read pairs (2 x 75 bp)	Mapped reads	% mapped
WT	146,560,220	121,413,511	83%
WT 0u 5-aza-2dC rep1	137,038,933	105,153,917	77%
WT 0u 5-aza-2dC rep2	139,474,312	107,636,475	77%
WT 4u 5-aza-2dC rep1	146,601,538	110,413,816	75%
WT 4u 5-aza-2dC rep2	139,171,755	104,978,343	75%
Del-hinge clone a	146,560,220	126,226,252	86%
Del-hinge clone b	153,499,202	115,995,076	76%
Del-hinge 0u 5-aza-2dC rep1	133,661,415	103,975,758	78%
Del-hinge 0u 5-aza-2dC rep2	126,077,695	99,068,067	79%
Del-hinge 4u 5-aza-2dC rep1	129,875,455	102,072,146	79%
Del-hinge 4u 5-aza-2dC rep2	121,252,477	94,826,906	78%
Del-Ds-TR	148,259,851	125,850,134	85%
Del-Dxz4	137,042,219	116,563,384	85%
Inv_Dxz4 clone a	80,967,254	54,727,362	68%
Inv_Dxz4 clone b	75,241,635	49,830,943	66%
Wild-type Patski2-4 clone rep1	85,856,348	55,750,224	65%
Wild-type Patski2-4 clone rep2	94,294,601	61,415,780	65%

Total RNA-seq reads, mapped reads and mapped percentage for each library.

Supplementary Table 7: Summary of differential expression results for various sample pairs

Comparison	Autosomes			chrX			See also
	Diploid	*spretus*	BL6	Diploid	*spretus*	BL6	
Del-hinge clone a vs. WT	2,689	1,634	1,552	80	55	16	Supplementary Data 2, 3
Inv-Dxz4 clone a vs. WT	2,049	1,237	983	86	48	17	
Inv-Dxz4 clone a vs. Del-hinge clone a	2,967	2,196	2,211	105	75	25	
WT 4u-5aza-2dC vs. WT 0u-5aza-2dC	2,968	1,591	1,640	156	42	48	Supplementary Data 6
Del-hinge 4u-5aza-2dC vs. Del-hinge 0u-5aza-2dC	1,451	727	694	75	12	33	Supplementary Data 7
Del-hinge 4u-5aza-2dC vs.WT 4u-5aza-2dC	4,710	2,726	3,578	197	102	53	
Patski2-4 vs. WT	2,998	2,253	2,817	115	81	57	
Patski2-4 vs. WT 0u-5aza-2dC	4,350	3,428	3,872	184	128	49	
Patski2-4 vs. WT 4u-5aza-2dC	5,738	4,057	4,433	266	140	67	
Patski2-4 vs. Del1-hinge clone a	5,073	3,984	4,176	185	135	42	
Patski2-4 vs. Del-hinge 0u-5aza-2dC	5,540	4,340	4,509	198	143	54	
Patski2-4 vs. Del-hinge 4u-5aza-2dC	5,942	4,405	4,555	233	133	60	
Patski2-4 vs. InvDxz4 clone a	317	199	262	8	4	4	Supplementary Data 5

Counts of differentially expressed autosomal and X-linked genes (both diploid and allelic) between relevant samples where replicates were available.

Supplementary Table 8. List of sgRNAs for CRISP/Cas9 editing

sgRNA_ID	Sequence	Genomic location (mm10)
Ds1*	GTGGTTAGGGACTTTCATAGGGG	chrX:75,637,503-75,637,525
Ds2	TGGCCACTGGGTA(g)GCGACAGCGG(a)	chrX:75,674,020-75,674,042
Ds3	AGGGATATTTTGGCTTCTTGTGG(t)	chrX:75,674,935-75,674,957
Dx1	GCCCCTCTAAATACCTAGTGCG(a)G	chrX:75,721,083-75,721,105
Dx2	GAGCAATGGGTCTGGGAAAGCGG(a)	chrX:75,764,738-75,764,760

*sgRNAs designed by CHOPCHOP were selected to include BL6 SNPs at the PAM site (red) if available. The *spretus* SNPs are listed as small letters in parenthesis. A pair of sgRNAs were used to edit each target: Ds-1&2 for Del-Ds-TR, Ds-2&3 for Inv-5' Ds-TR (CTCF peak inversion (chrX:75674066-75674317) at the 5' of Ds-TR, Dx1&2 for Del-Dxz4 and Inv-Dxz4, and Ds1&Dx2 for Del-hinge. See also Supplementary Fig. 1a.

Supplementary Table 9. List of primers

Primer	Sequence	Note
Ds_F1*	TCATCAACGCGATGAGAGAC	Primer pair flanking the Ds-TR_cut1 site
Ds_R1	CACATCCTTGCACTGCATCT	
Ds_F2	TGAGCCAGAGAACCAGGAGT	Primer pair flanking the Ds-TR_cut2 site
Ds_R2	TGACAGACAGGAAGTGCGTC	
Ds_F3	GACGCACTTCCTGTCTGTCA	Primer pair flanking the Ds-TR_cut3 site
Ds_R3	CAGAGCACTTGGTCGAAACA	
Dx_F1	GACTAACACAGAAGCGGTCCTT	Primer pair flanking the Dxz4_cut1 site
Dx_R1	TTTATGTGCTTCGGTGTAATGC	
Dx_F2	TTCCGCATCTCAGAAGGAGT	Primer pair flanking the Dxz4_cut2 site
Dx_R2	CAGGCAAAACATCCACAATG	
Dxz4_ChIP_F	AACAAAAGCAAGGGGAACCT	Primers for Dxz4 repeats
Dxz4_ChIP_R	CAGGTCCTGAGCACAGTTCA	
H19_ChIP_F	GGGTAGCTCCTTCAGTCTTGCGCCCTTCAC	Primers for H19 imprinting control region
H19_ChIP_R	GCACAAATGCCTGATCCCTTTGTTGAACCTGG	
18S_F	TGAGGCCATGATTAAGAGGG	Primers for 18S RT-PCR
18S_R	AGTCGGCATCGTTTATGGTC	
ActB_F	GCTCTTTTCCAGCCTTCCTT	Primers for ActB RT-PCR
ActB_R	CTTCTGCATCCTGTCAGCAA	
Xist_F	TGCCCTTTCTCAATTCCATC	Primers for Xist RT-PCR
Xist_R	GGCCTGCTTTTGCTATTCAG	

*Five different combinations of primer pairs were used for each pair of CRISPR/Cas9 cutting sites to verify deletion or inversion of loci. For example, for Dxz4 cut1 and 2, Dx_F1/R1, Dx_F2/R2, Dx_F1/R2 were used to verify deletion, and Dx_F1/F2 to verify inversion (see also Supplementary Fig. 1a,b). Primers to verify the efficiency of CTCF ChIP for Dxz4 and H19 as well as primers used for RT-PCR are also listed.
