# Supplementary material for: Orientation-dependent Dxz4 contacts shape the 3D structure of the inactive X chromosome
Source: Nat Commun. 2018 Apr 13;9:1445. doi: 10.1038/s41467-018-03694-y (PMC5899087; doi:10.1038/s41467-018-03694-y)
Supplement: Supplementary file 2 — Description of Additional Supplementary Files [file 41467_2018_3694_MOESM2_ESM.pdf]

Description of Additional Supplementary Files

File name: Supplementary Data 1
Description: Allelic read counts per chromosome. Allelic read counts by chromosome and genome-wide along with the allelic ratio (BL6/*spretus*) and the ratio relative to the genome-wide ratio for genomic DNA (Patski wild-type), ChIP input (Patski wild-type and Del-hinge) and in-situ DNase Hi-C libraries (Patski wild-type, Del-hinge, and Inv-Dxz4). For in-situ DNase Hi-C libraries, tables were generated for (1) all read pairs (cis+trans), (2) interchromosomal read pairs (trans only), (3) intrachromosomal reads pairs (cis only), and (4) intrachromosomal reads pairs separated by at least 20kb (cis > 20kb). Note that for (1) and (2) counts are based on each end of filtered and segregated read pairs, while for (3) and (4) counts are based on filtered and segregated read pairs. Genomic DNA was sequenced as 36bp single-end reads, ChIP input as 75bp paired-end reads mapped and segregated jointly (see Supplementary Table 4), and in-situ DNase Hi-C as 80 - 150bp paired-end reads mapped and segregated separately and combined as valid read pairs (see Supplementary Table 1).

File name: Supplementary Data 2
Description: Differential Xi-specific expression changes between Del-hinge clone a and WT. DESeq2 differential expression results between Del-hinge clone a and WT for each X-linked gene. Columns give the mean expression, log2 fold change in expression, p-value from the Wald test, and p-value adjusted to account for multiple testing. Highlighted genes show a significant differential expression (|log fold change| >= 0.5 and adjusted p-value <=0.05 by the Wald test). The additional columns give the Xi- and Xa-specific TPM values for each of the replicates used in the differential analysis.

File name: Supplementary Data 3
Description: Differentially expressed autosomal genes between Del-hinge clone a and WT. As described in Supplementary Data 2, but for 2689 significantly differentially expressed autosomal genes based on biallelic expression. Sorted by chromosomal position.

File name: Supplementary Data 4
Description: Genes called escape in Patski WT lines**.** The mm10 genes listed were classified as genes that escape XCI in 3/3 WT lines based on criteria described in the text, except for *Ftx* classified as escape in 2/3 lines. For each line, the ratio between the number of reads from the Xi versus the total number of reads assigned either to the Xi or Xa is listed, together with the number of reads containing SNPs specific for the Xi or Xa, the total TPM, the Xi- and Xa- specific TPM, and the lower and upper confidence limits of escape probability (see methods).

File name: Supplementary Data 5
Description: Differential Xi-specific expression changes between Inv-Dxz4 and Patski2-4 wild-type. As described in Supplementary Data 2, but for Inv-Dxz4 and Patski2-4 WT.

File name: Supplementary Data 6
Description: Differential Xi-specific expression changes between 5-aza-2dC treated and untreated WT. As described in Supplementary Data 2, but for 5-aza-2dC treated and untreated WT.

File name: Supplementary Data 7
Description: Differential Xi-specific expression changes between 5-aza-2dC treated and untreated Del-hinge clone a**.** As described in Supplementary Data 2, but for 5-aza-2dC treated and untreated Del-hinge clone a.
